# Supplementary figures and images for: Spatial modeling reveals nuclear phosphorylation and subcellular shuttling of YAP upon drug-induced liver injury
Source: eLife. 2022 Oct 18;11:e78540. doi: 10.7554/eLife.78540 (PMC9578710; doi:10.7554/eLife.78540)

Figure S1A supporting data

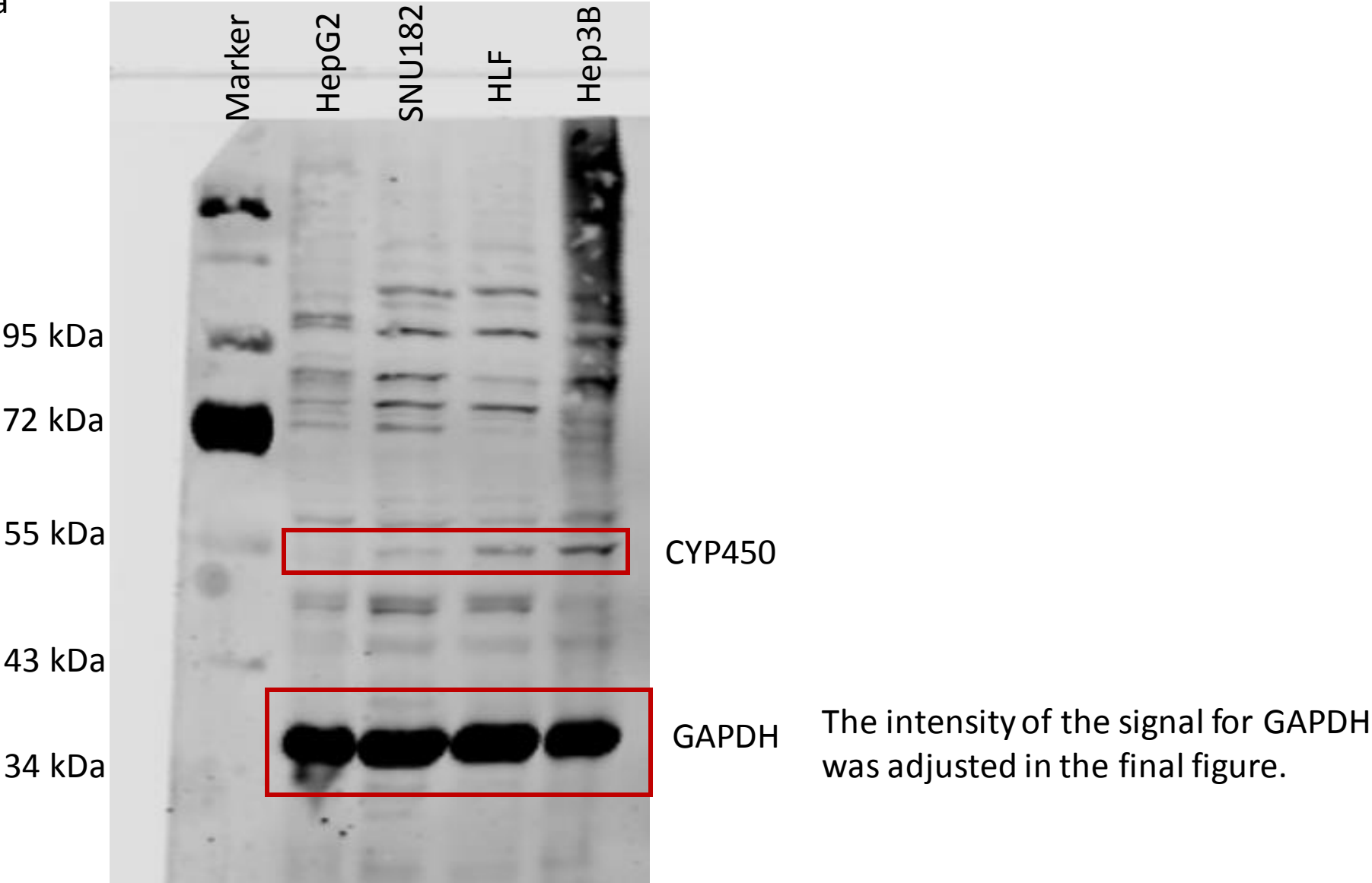

Supplement: Figure 1—figure supplement 1—source data 1. [file elife-78540-fig1-figsupp1-data1.zip › Figure 1-figure supplement 1-source data/Figure S1 source data.pdf]

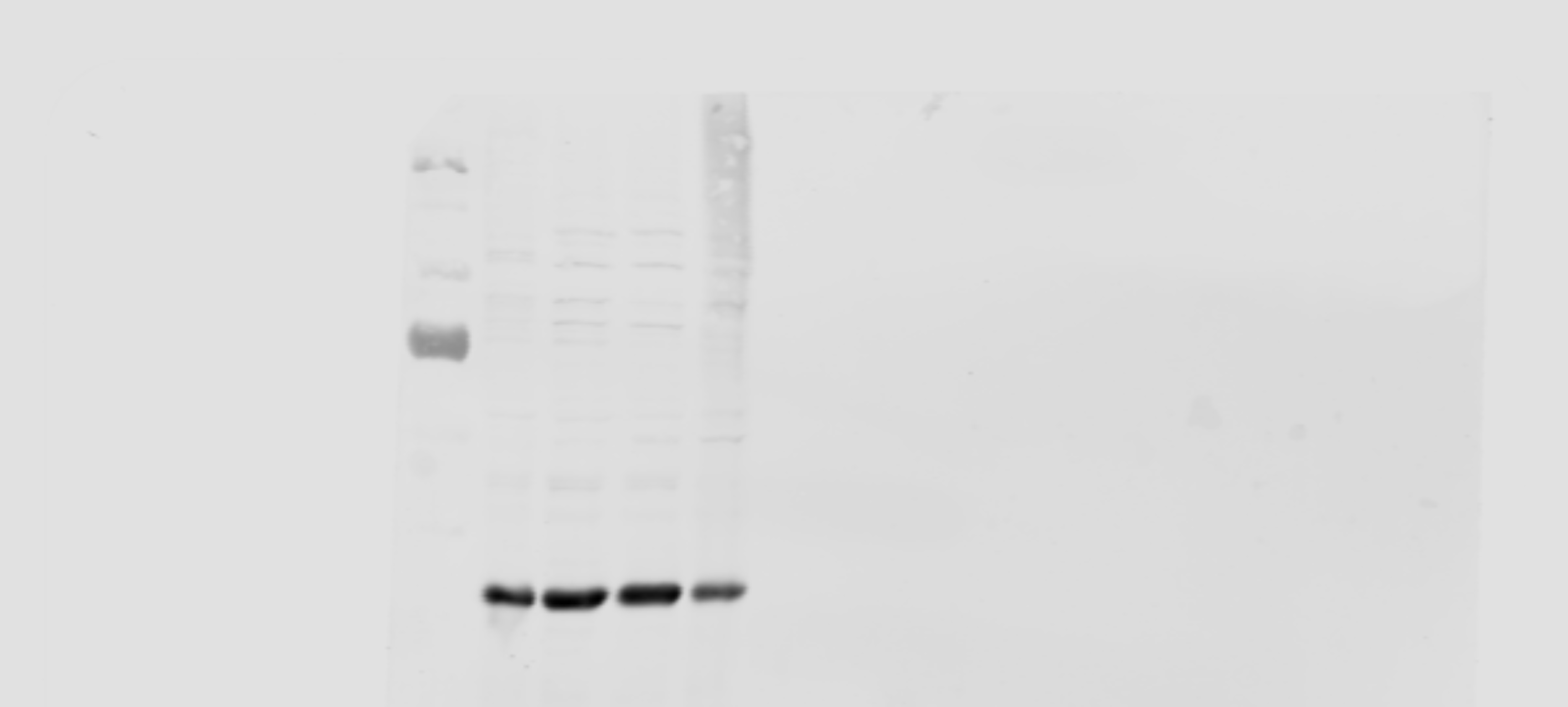

Supplement: Figure 1—figure supplement 1—source data 1. [file elife-78540-fig1-figsupp1-data1.zip › Figure 1-figure supplement 1-source data/Image_GAPDH.tif]

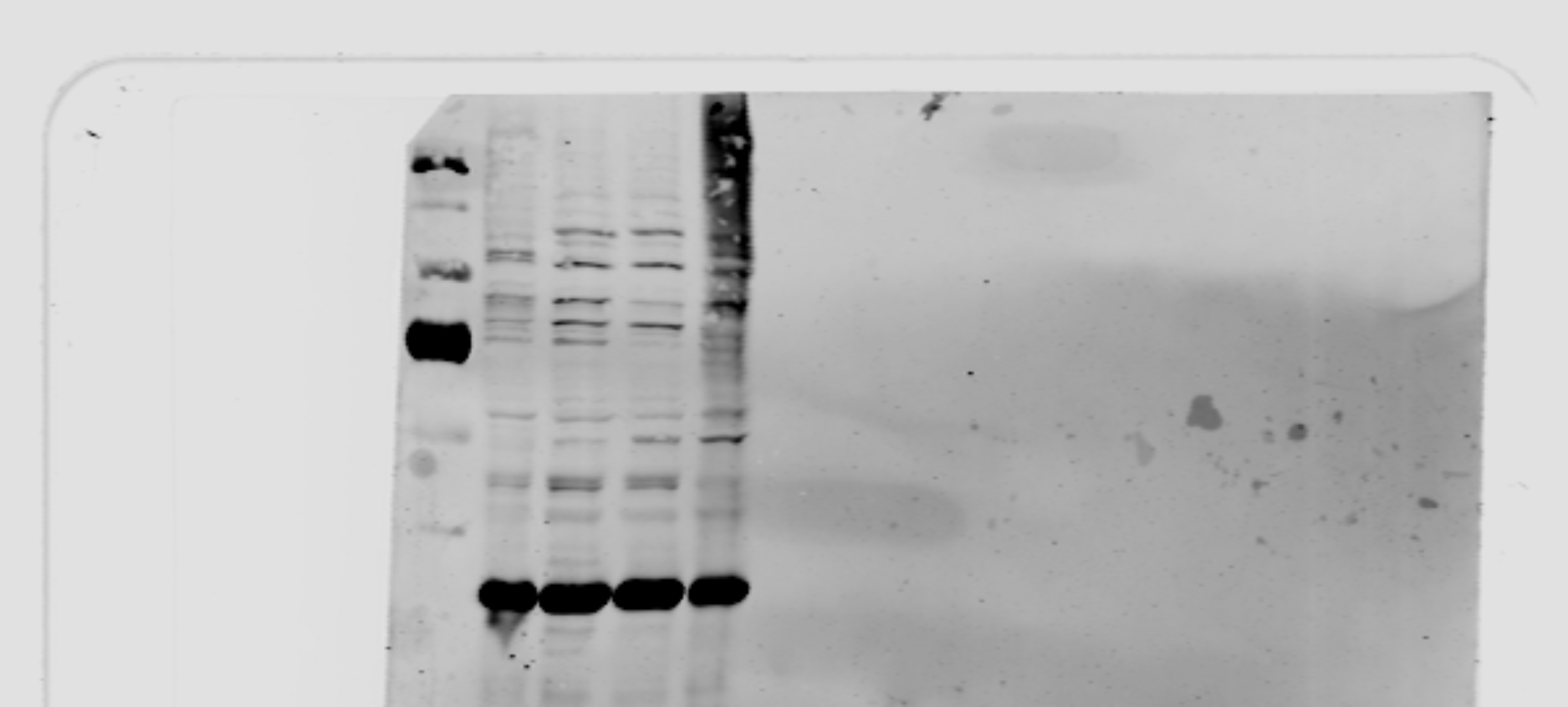

Supplement: Figure 1—figure supplement 1—source data 1. [file elife-78540-fig1-figsupp1-data1.zip › Figure 1-figure supplement 1-source data/Image_dark_CYP450 GAPDH.tif]

Figure 2 F

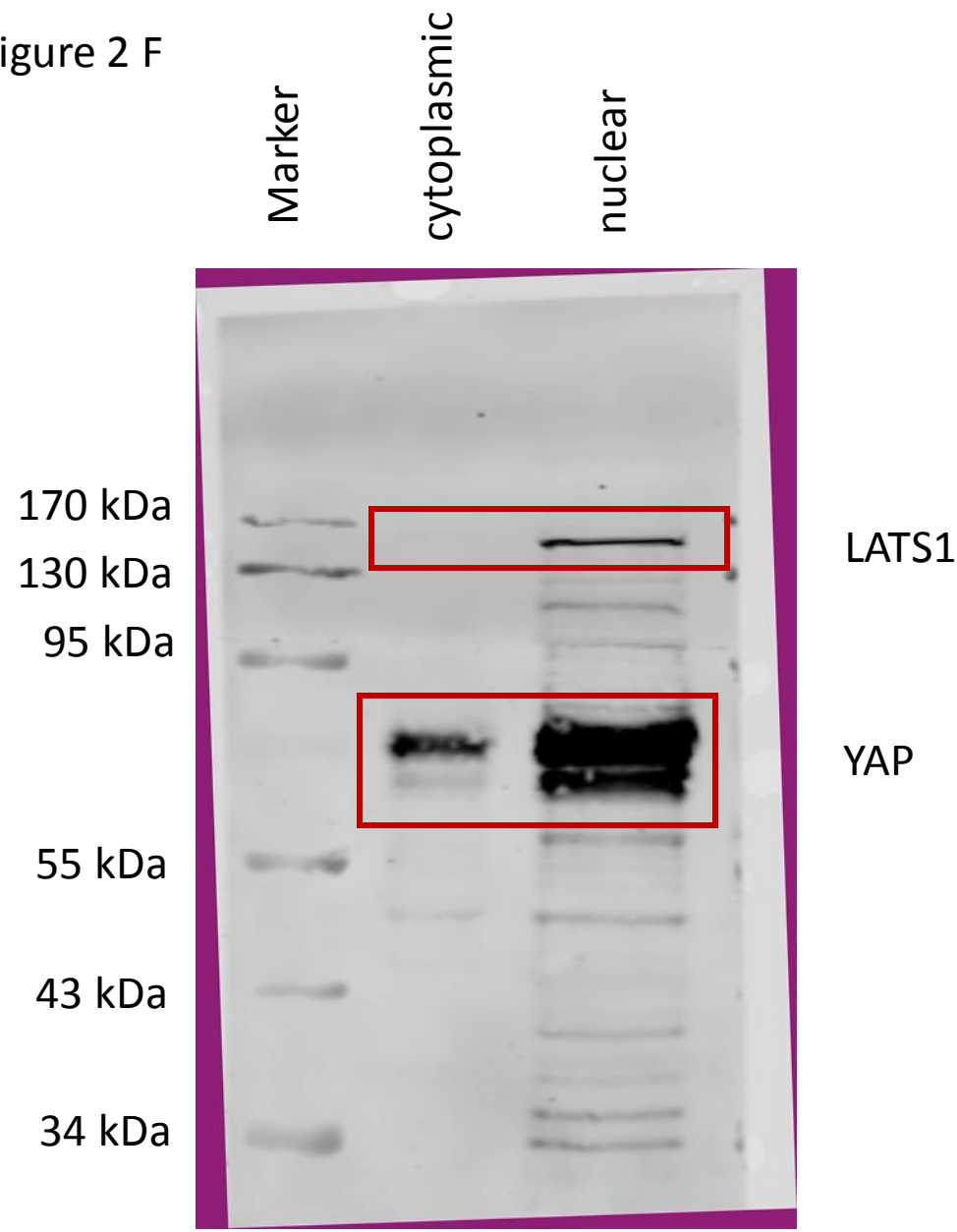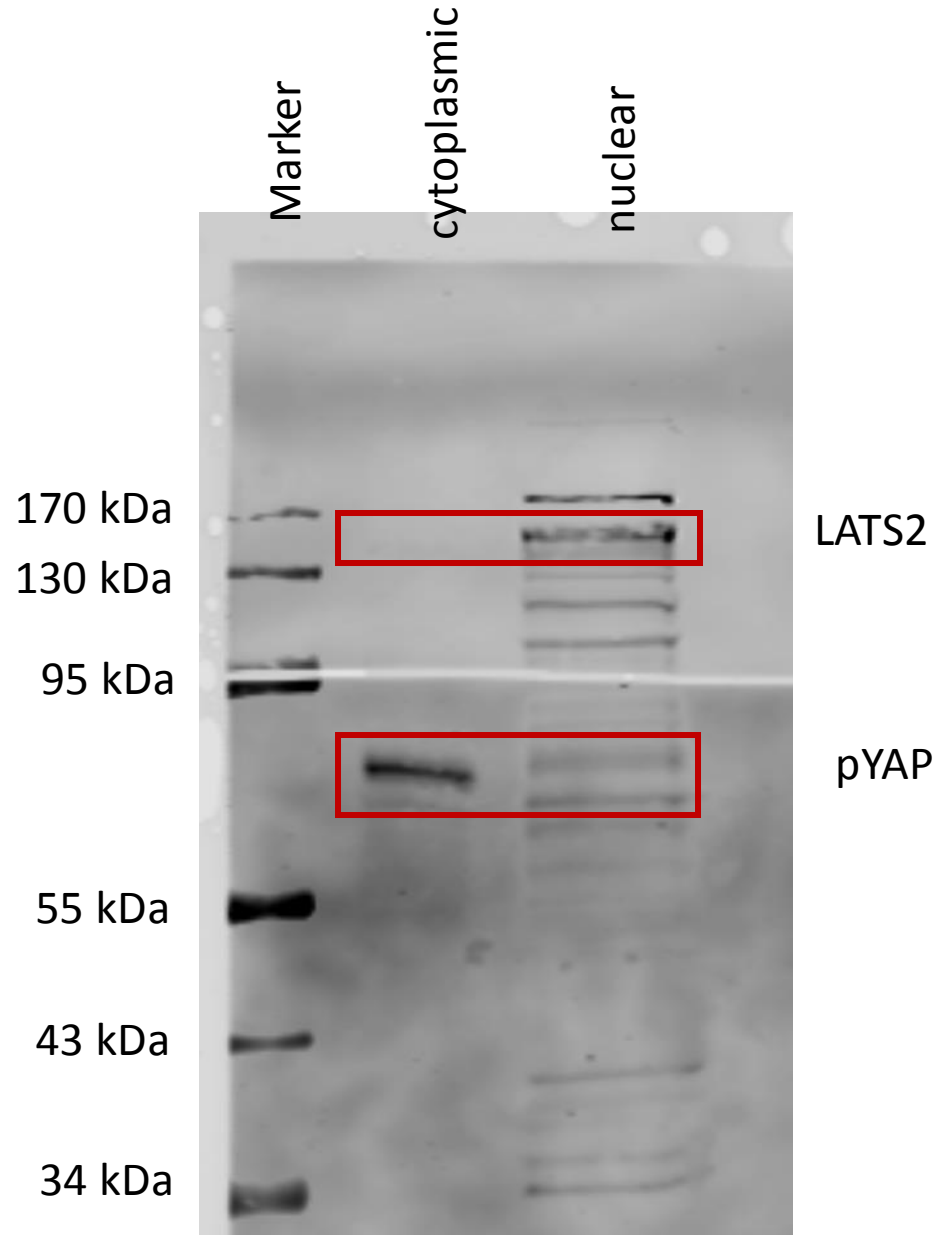

Figure 2 F

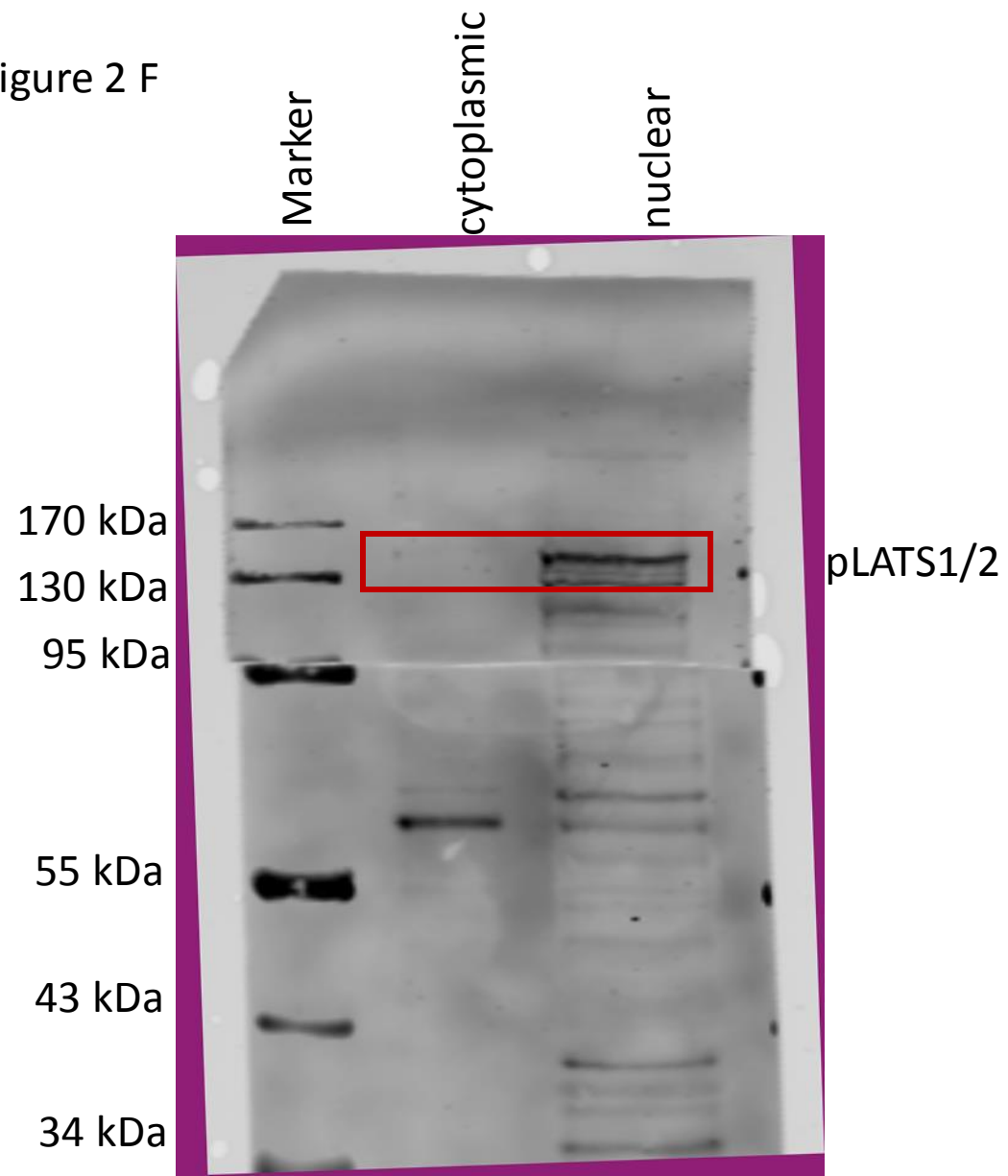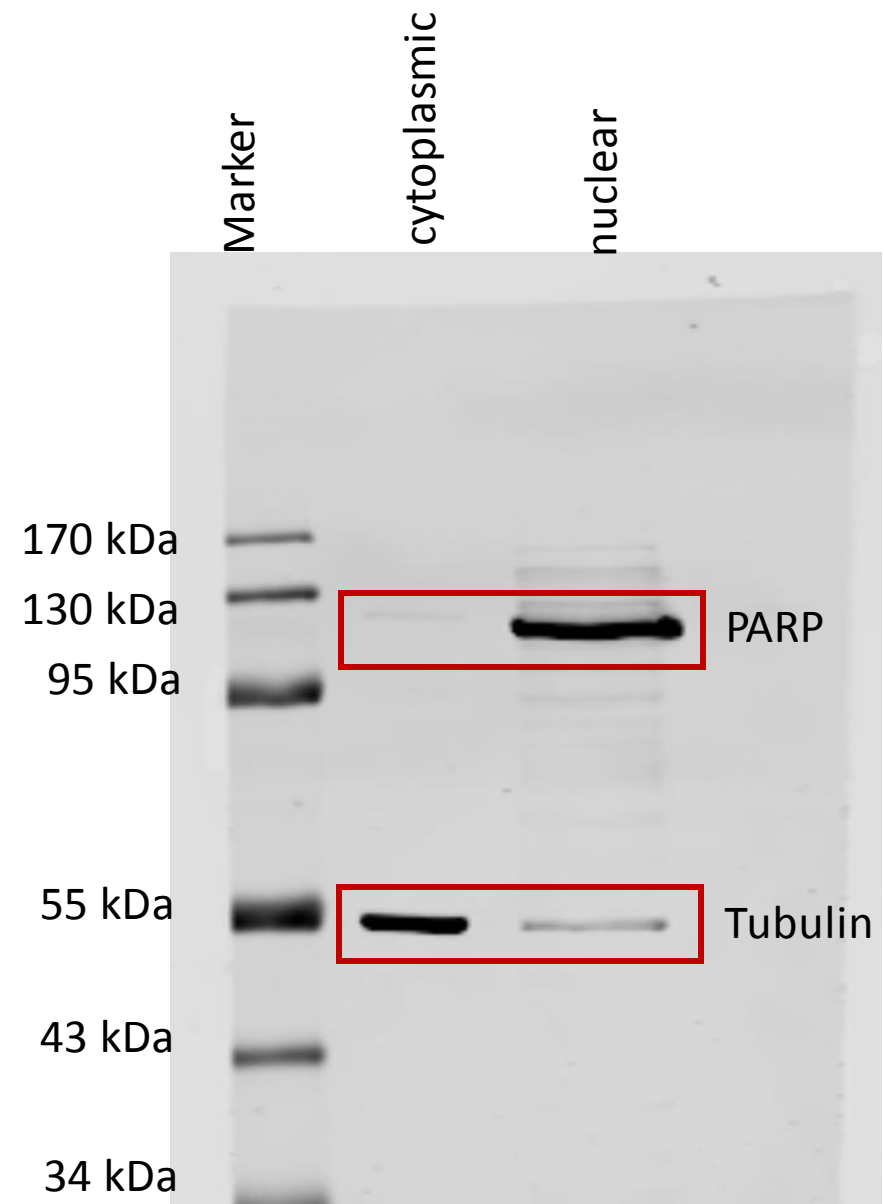

Supplement: Figure 2—source data 1. [file elife-78540-fig2-data1.zip › Figure 2-source data/Figure 2 source data.pdf]

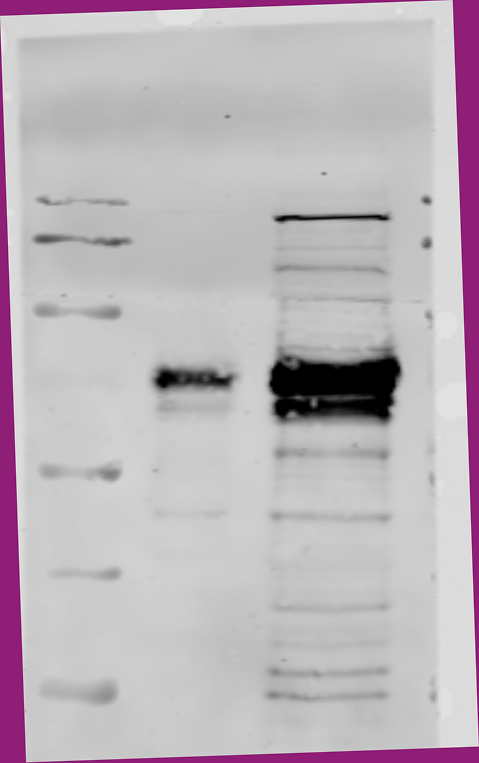

Supplement: Figure 2—source data 1. [file elife-78540-fig2-data1.zip › Figure 2-source data/Gel1.1 LATS1_ YAP.png]

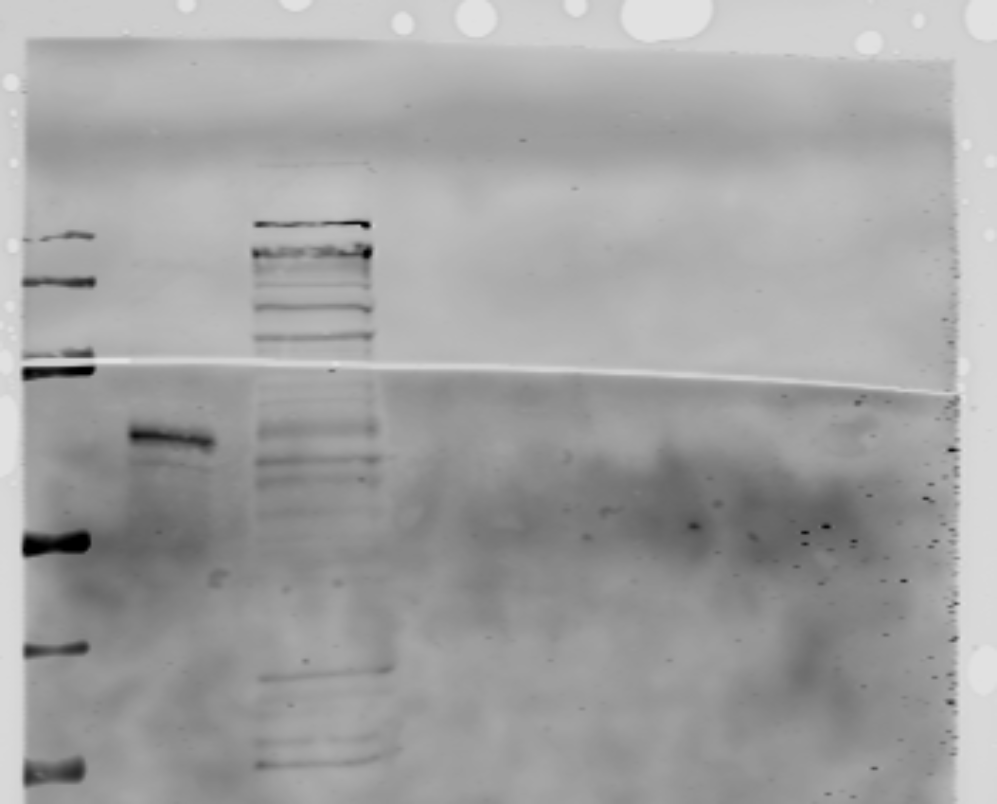

Supplement: Figure 2—source data 1. [file elife-78540-fig2-data1.zip › Figure 2-source data/Gel1.2 LATS2_ pYAP.png]

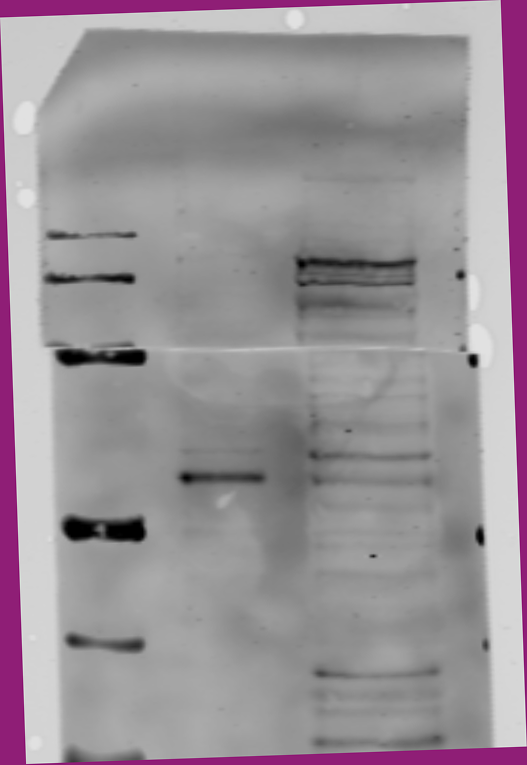

Supplement: Figure 2—source data 1. [file elife-78540-fig2-data1.zip › Figure 2-source data/Gel2.1p LATS1.png.png]

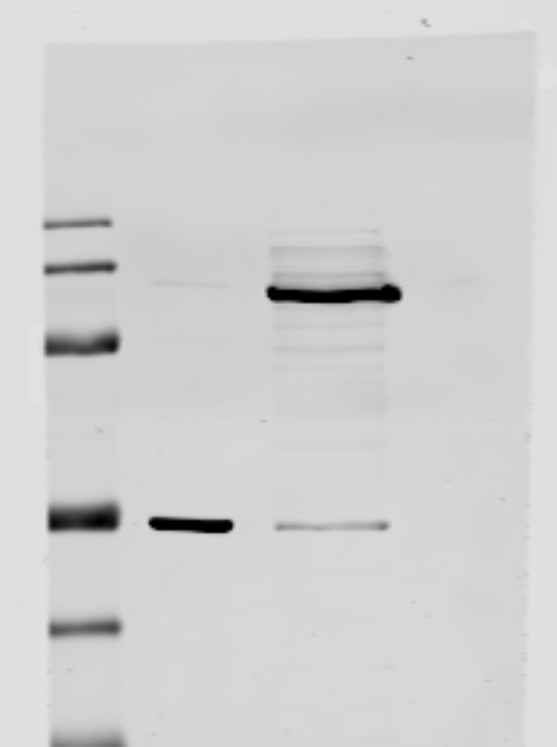

Supplement: Figure 2—source data 1. [file elife-78540-fig2-data1.zip › Figure 2-source data/Gel2.3 PARP_ Tubulin.png]

Fig 3C: 0.5 – 5 hours

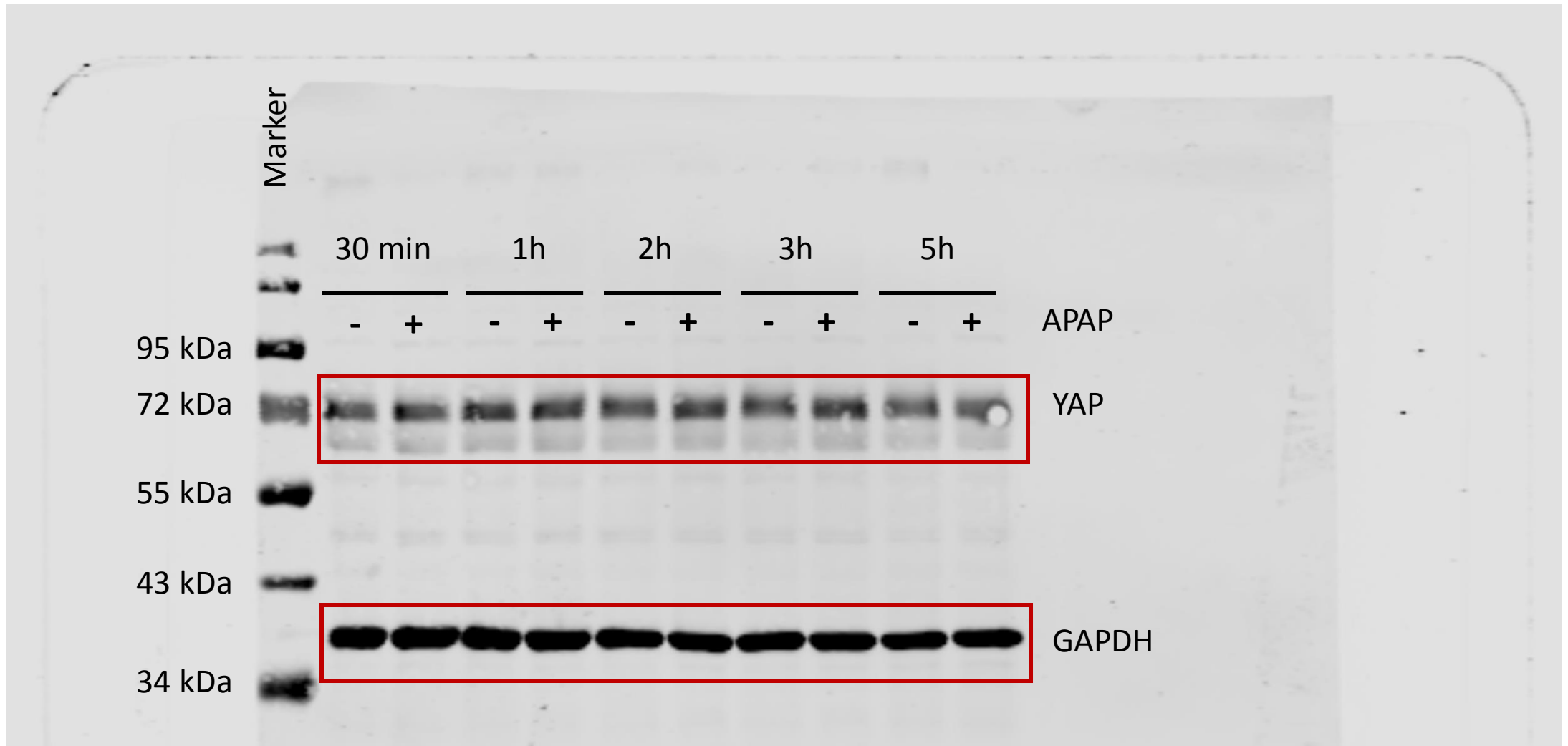

Fig 3C: 0.5 – 5 hours

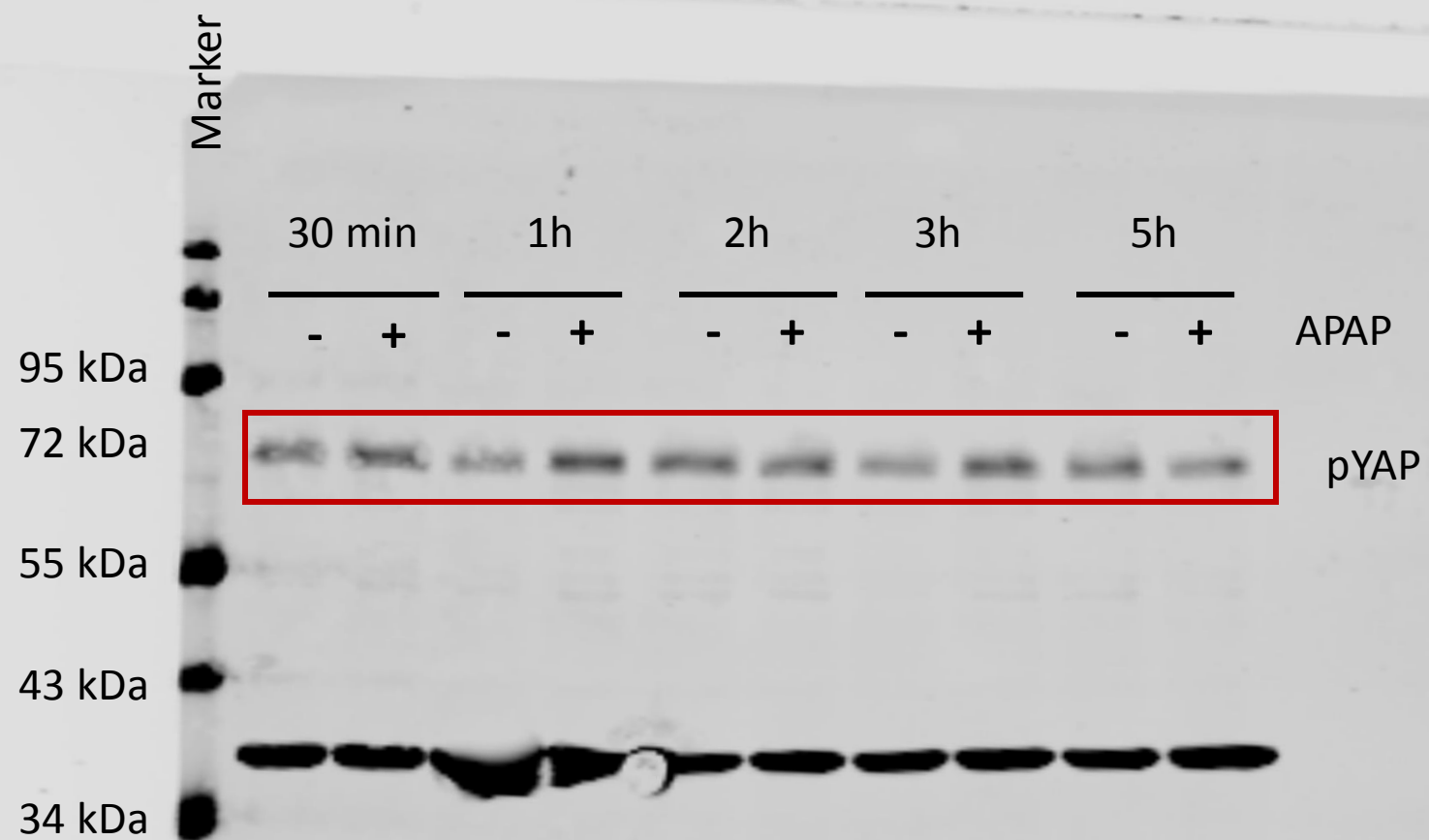

Fig 3C: 6 – 48 hours

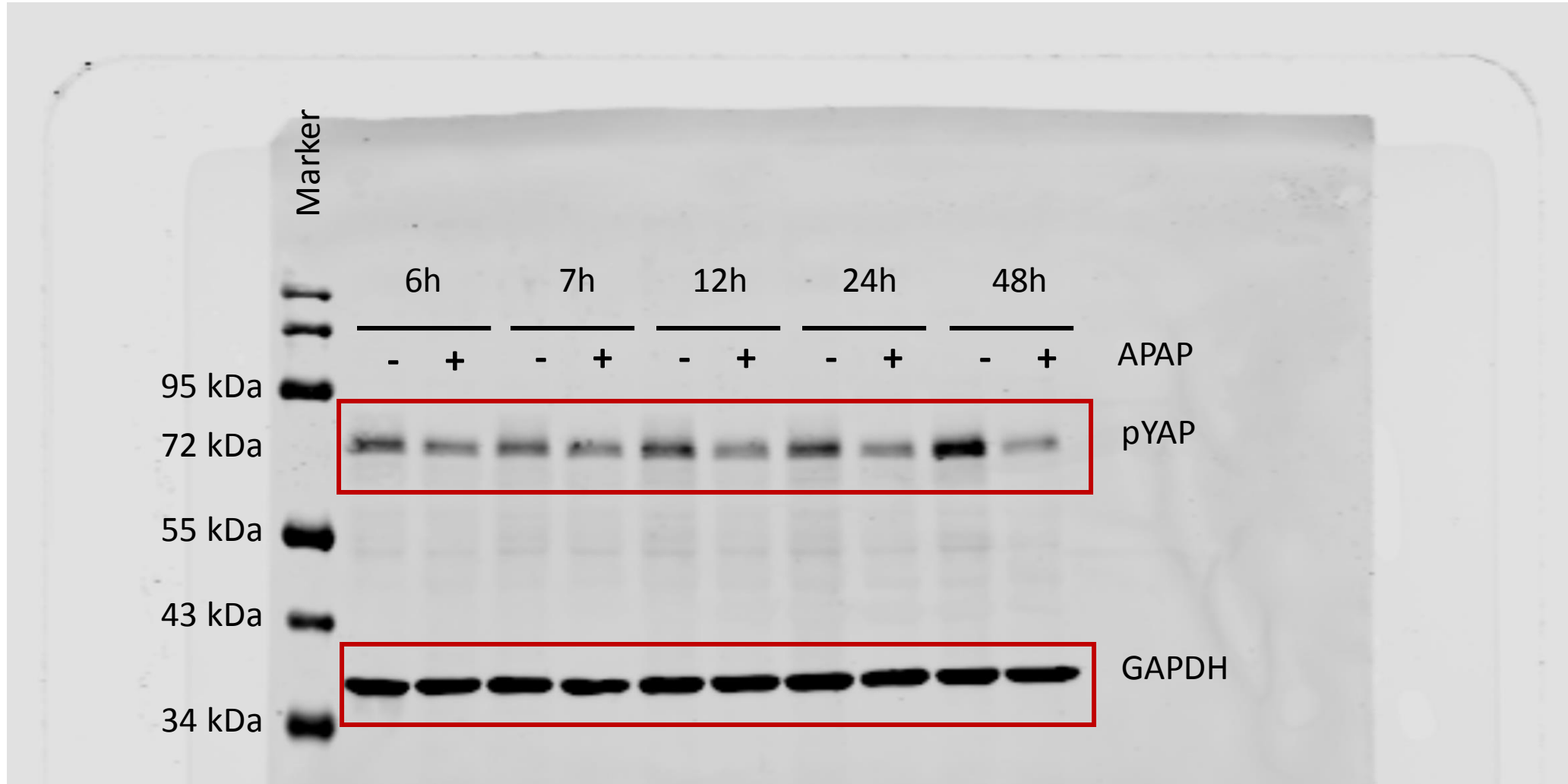

Fig 3C: 6 – 48 hours

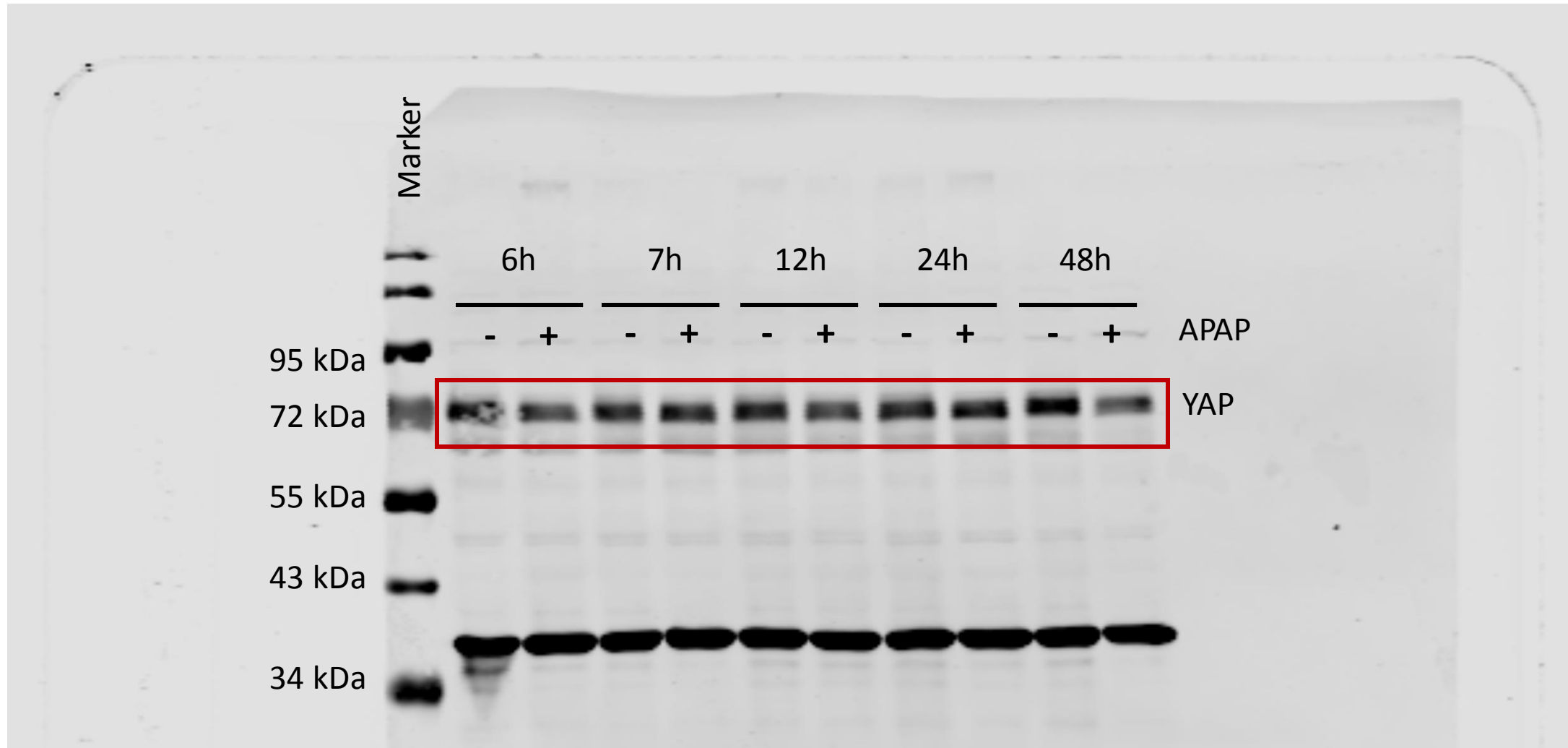

Supplement: Figure 3—source data 1. [file elife-78540-fig3-data1.zip › Figure 3-source data/Figure 3 - source data.pdf]

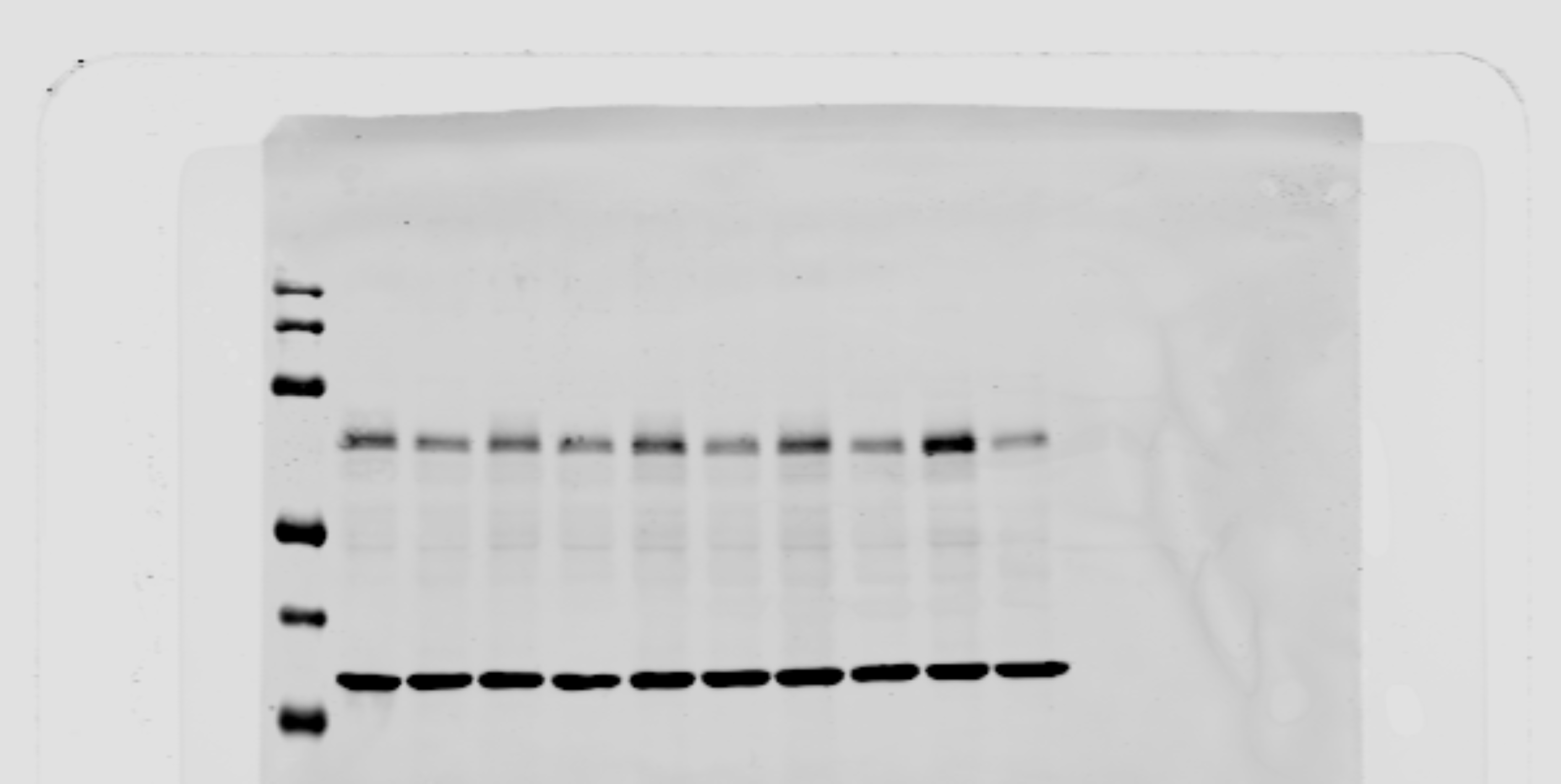

Supplement: Figure 3—source data 1. [file elife-78540-fig3-data1.zip › Figure 3-source data/Image_48h_pYAP GAPDH.tif]

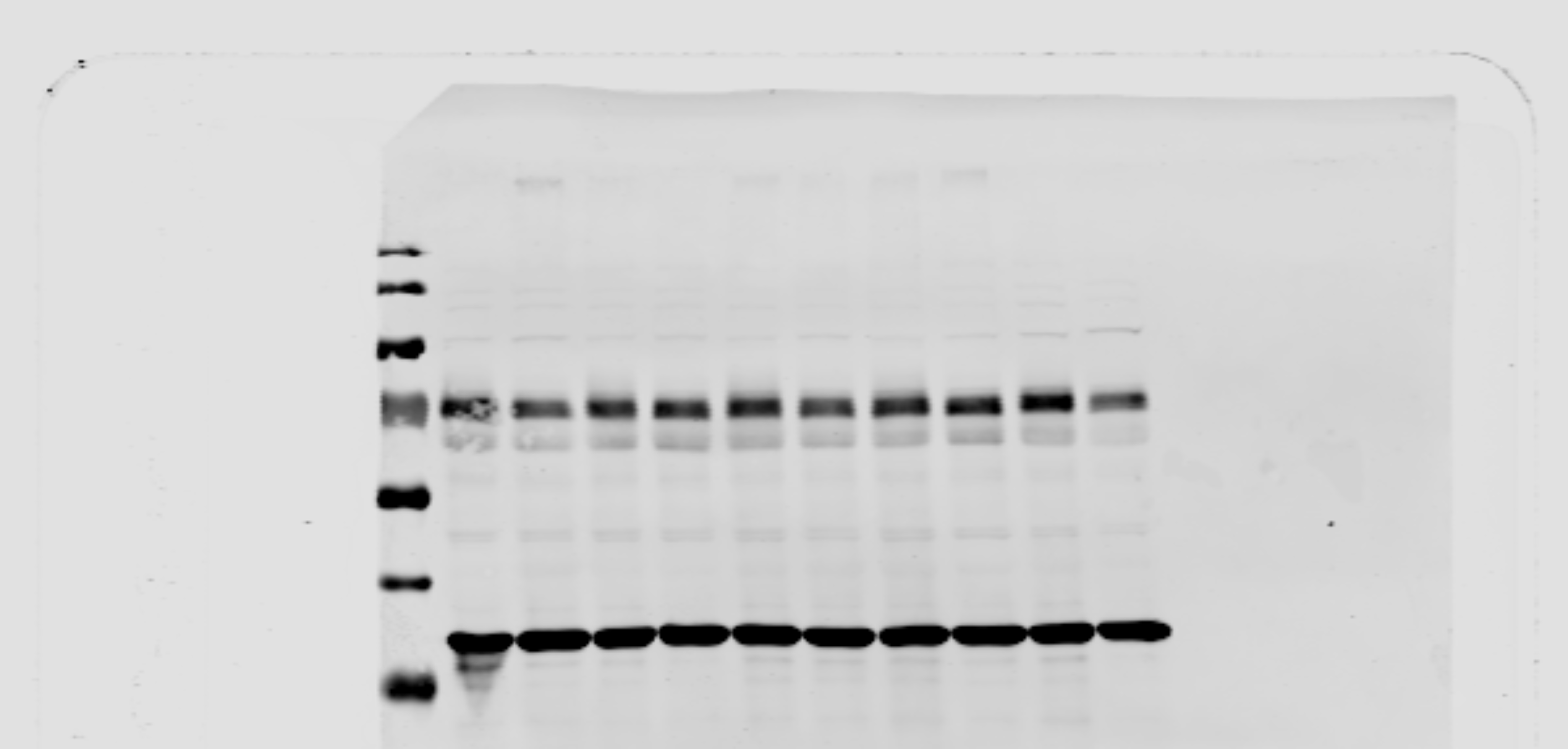

Supplement: Figure 3—source data 1. [file elife-78540-fig3-data1.zip › Figure 3-source data/Image_48h_YAP.tif]

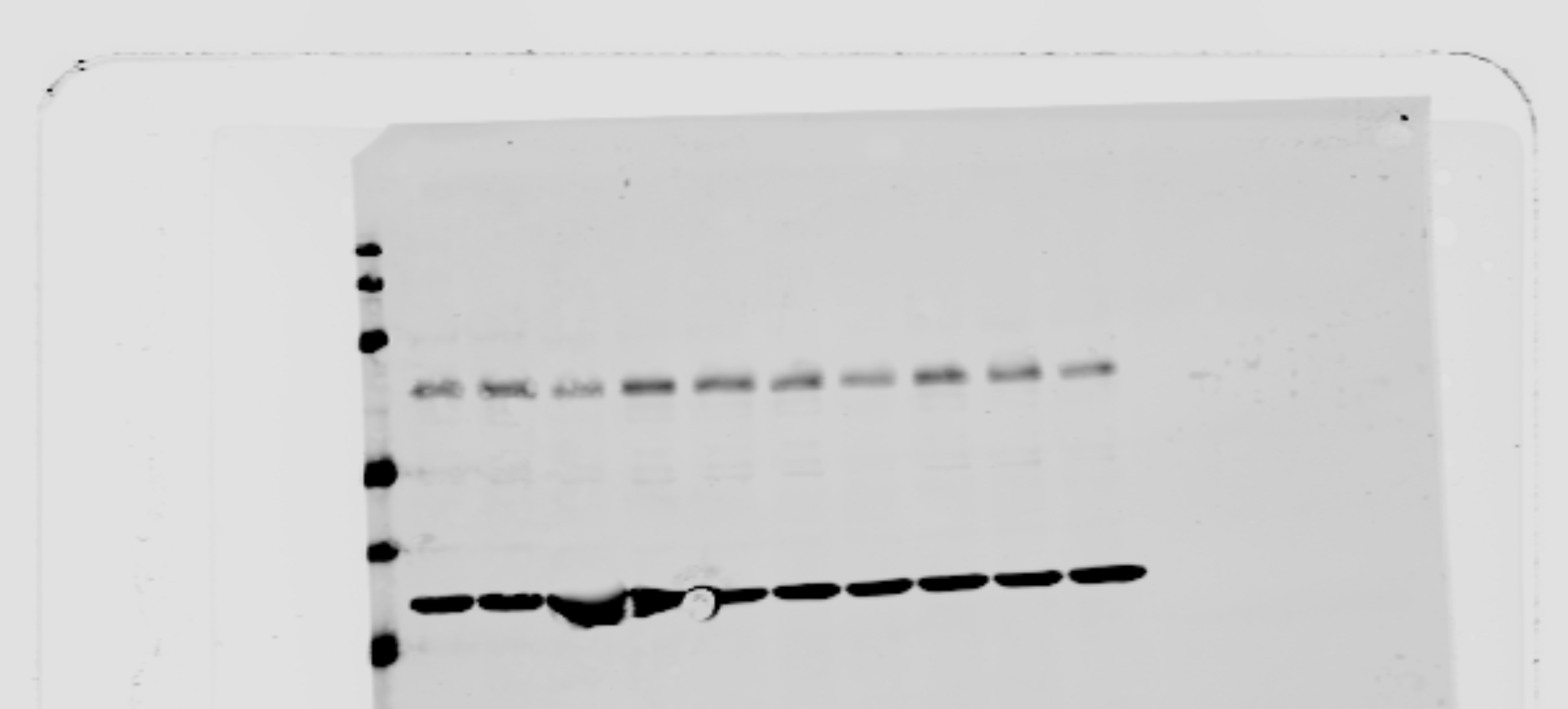

Supplement: Figure 3—source data 1. [file elife-78540-fig3-data1.zip › Figure 3-source data/Image_5h_pYAP.tif]

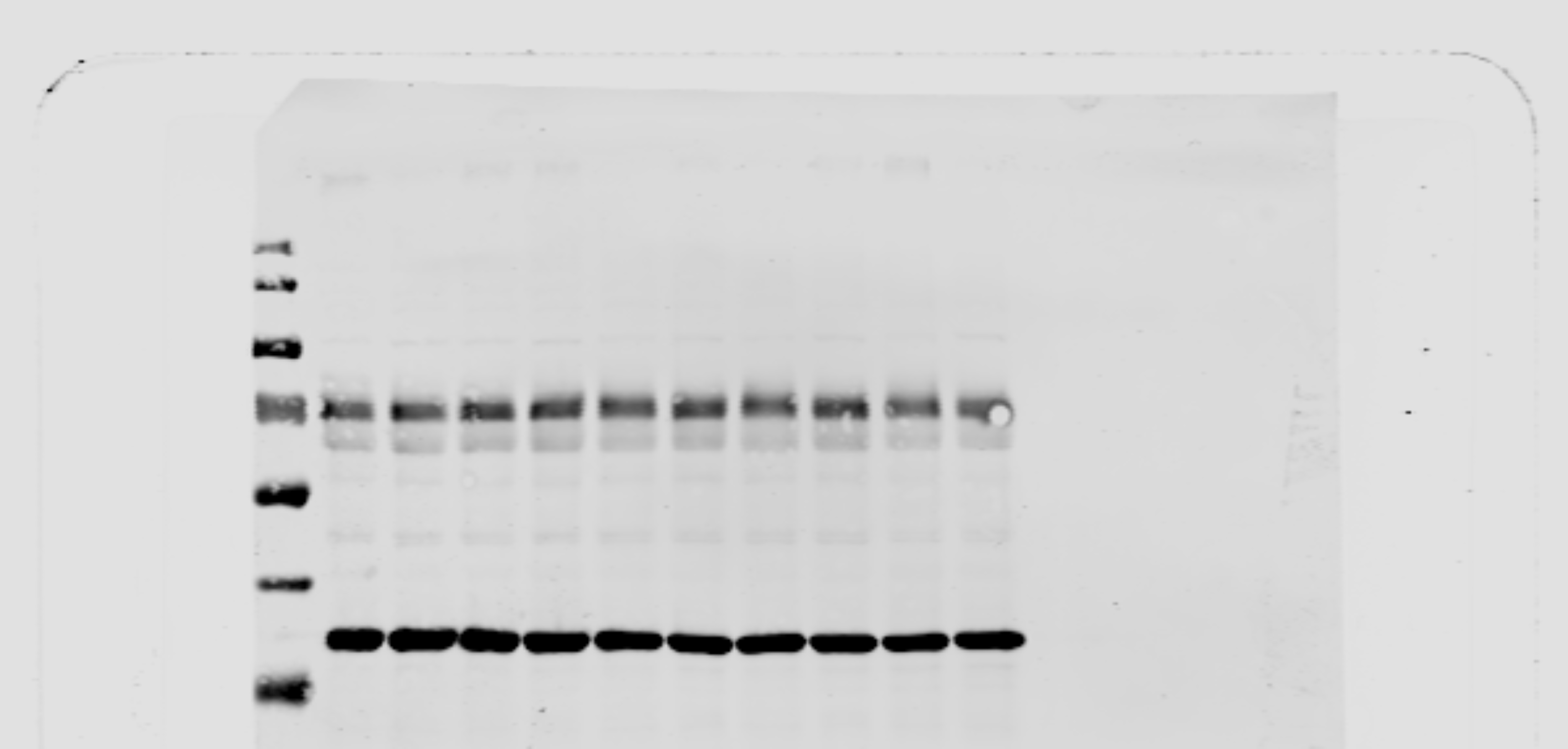

Supplement: Figure 3—source data 1. [file elife-78540-fig3-data1.zip › Figure 3-source data/Image_5h_YAP GAPDH.tif]

Figure S4 C

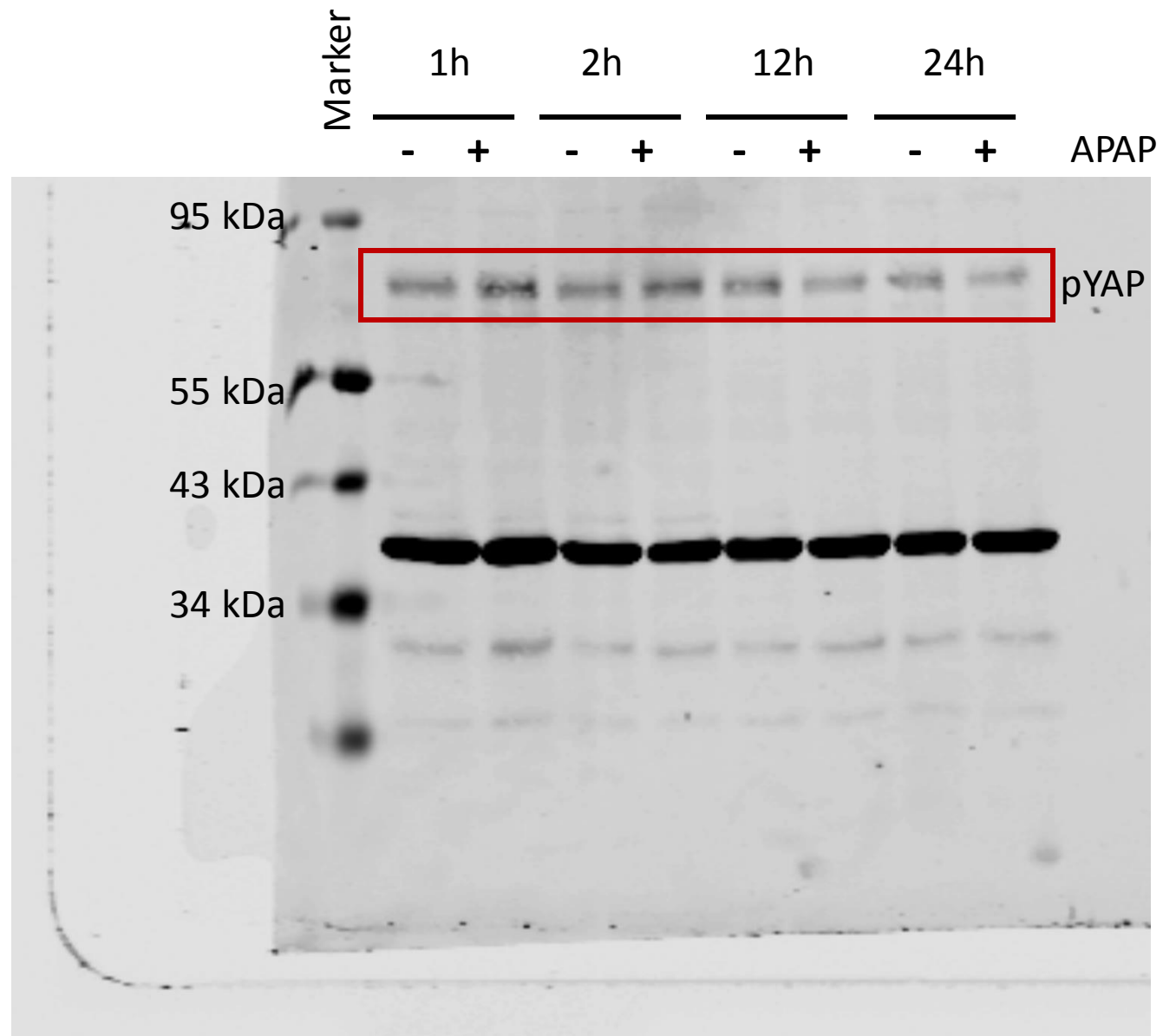

Figure S4 C

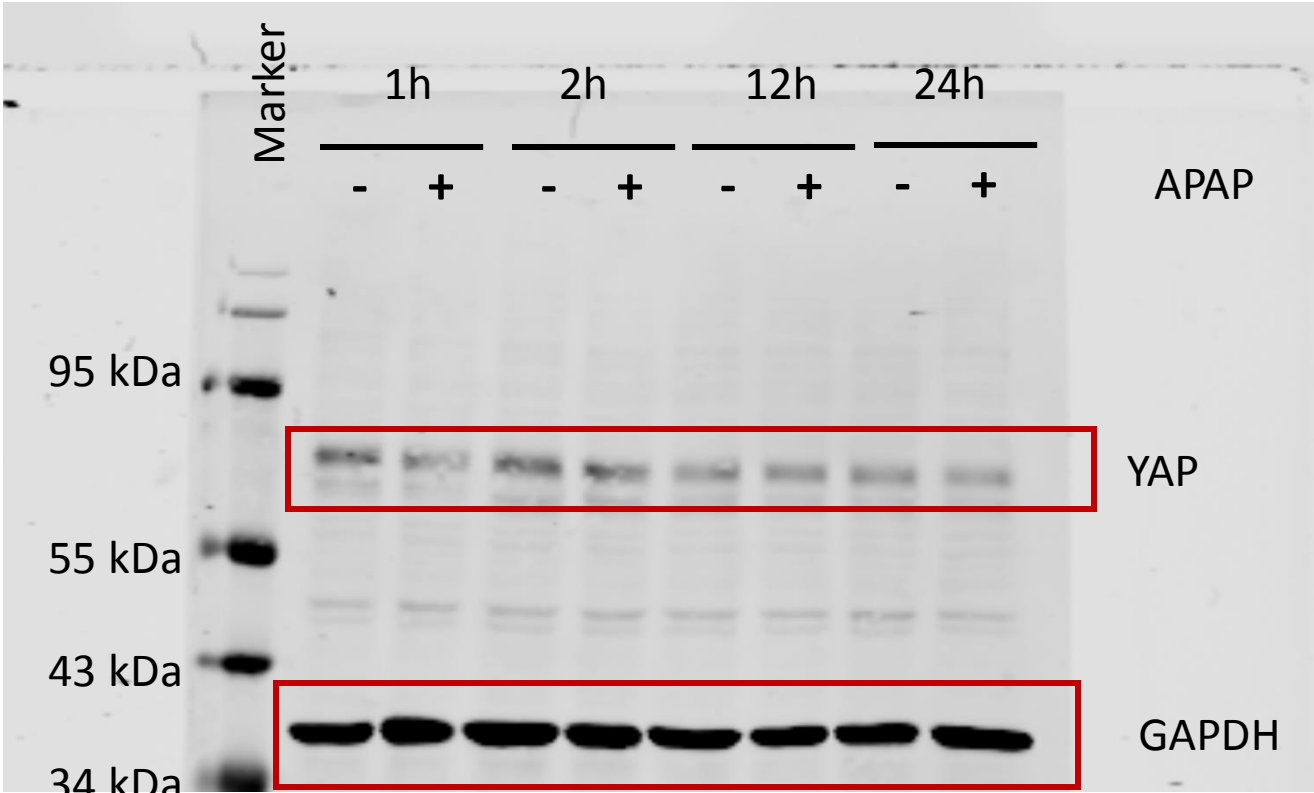

Supplement: Figure 3—figure supplement 1—source data 1. [file elife-78540-fig3-figsupp1-data1.zip › Figure 3-figure supplement 1-source data/Figure S4C Source data.pdf]

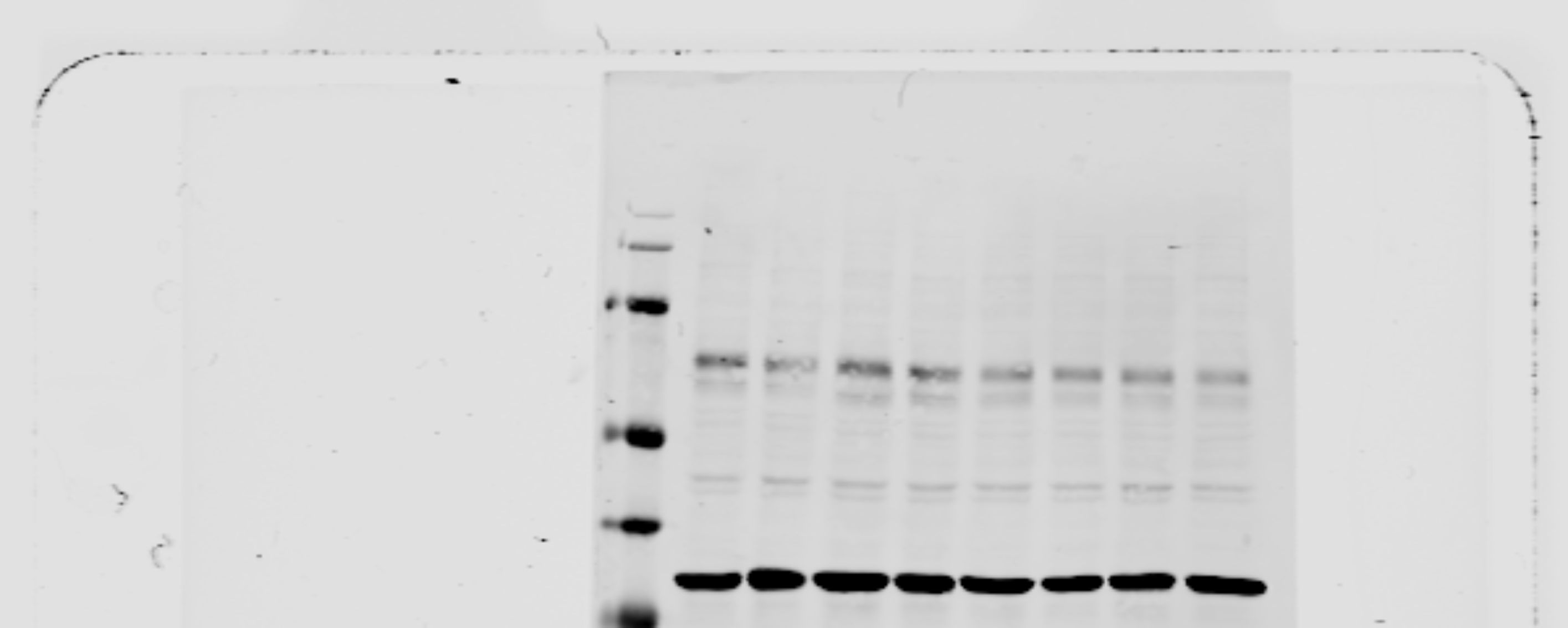

Supplement: Figure 3—figure supplement 1—source data 1. [file elife-78540-fig3-figsupp1-data1.zip › Figure 3-figure supplement 1-source data/Image_Jenny_YAP GAPDH.tif]

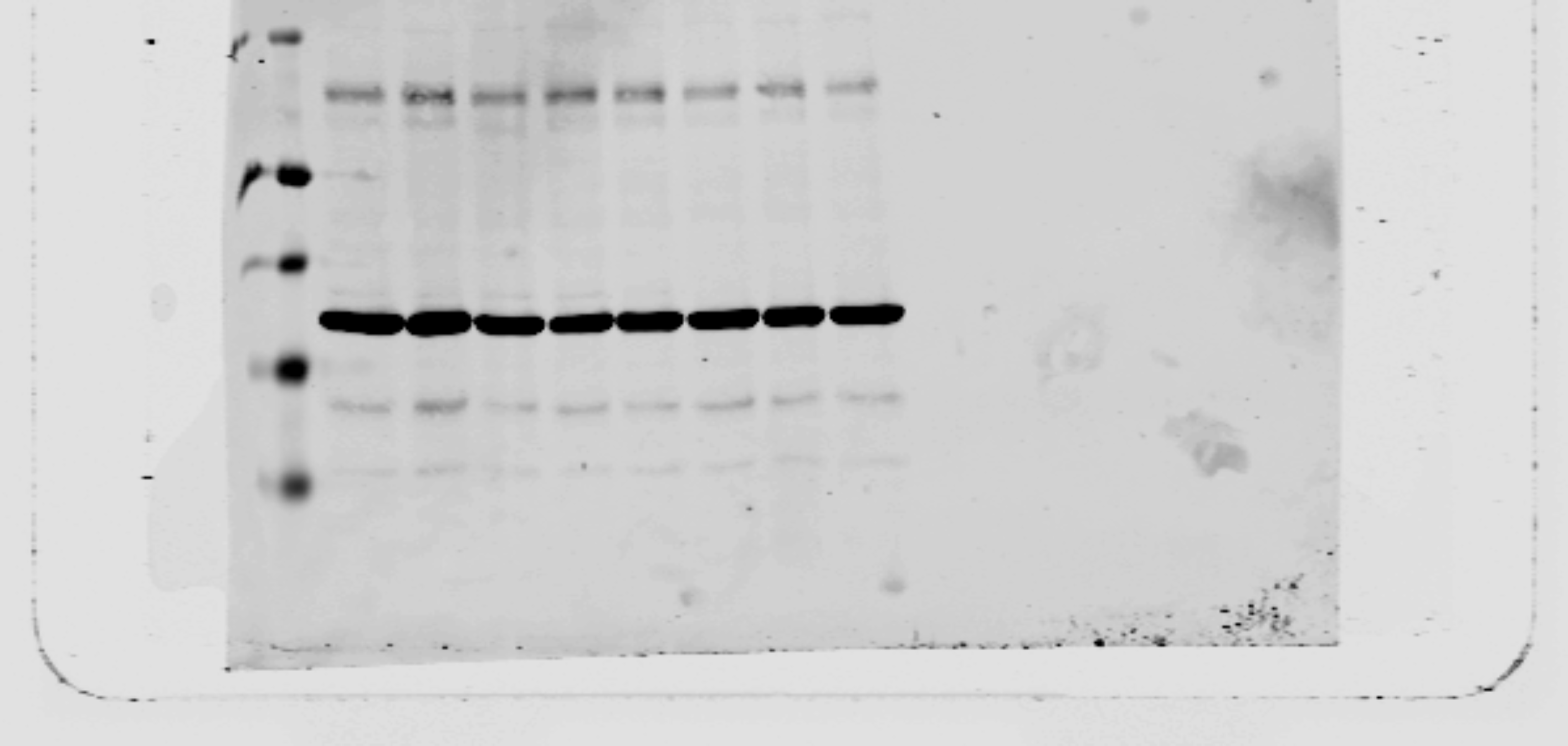

Supplement: Figure 3—figure supplement 1—source data 1. [file elife-78540-fig3-figsupp1-data1.zip › Figure 3-figure supplement 1-source data/pYAP.tif]

Fig 4 A (pYAP)

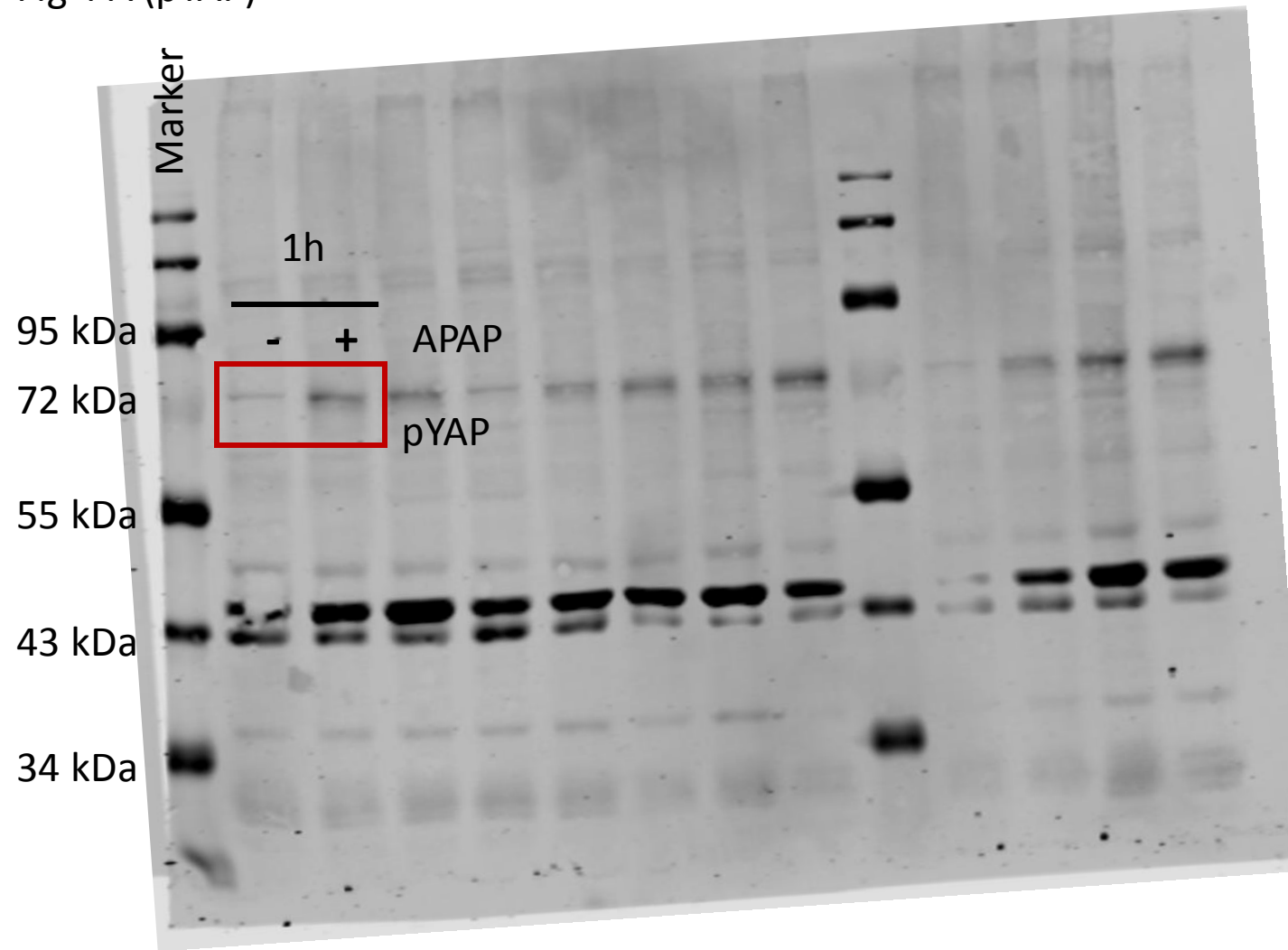

Fig 4 A (YAP)

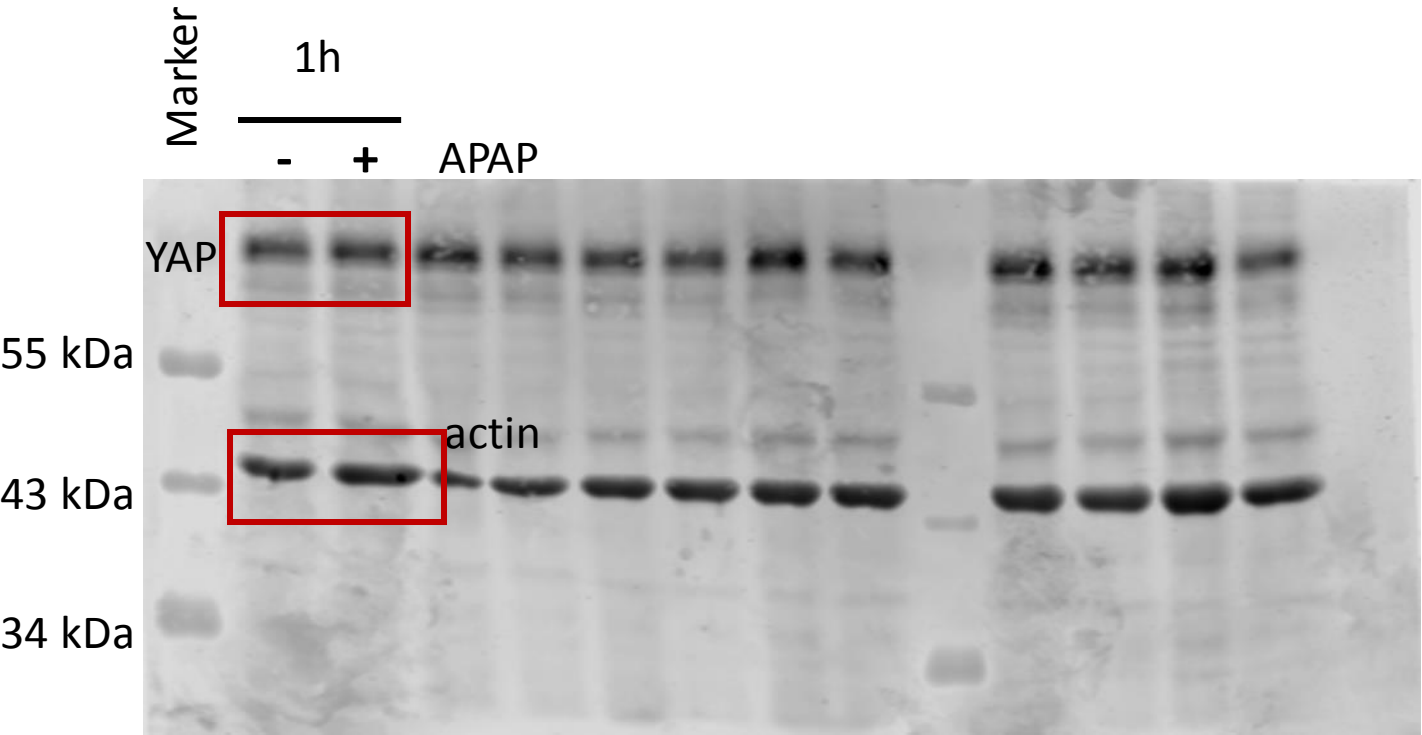

Fig 4 A (pAKT)

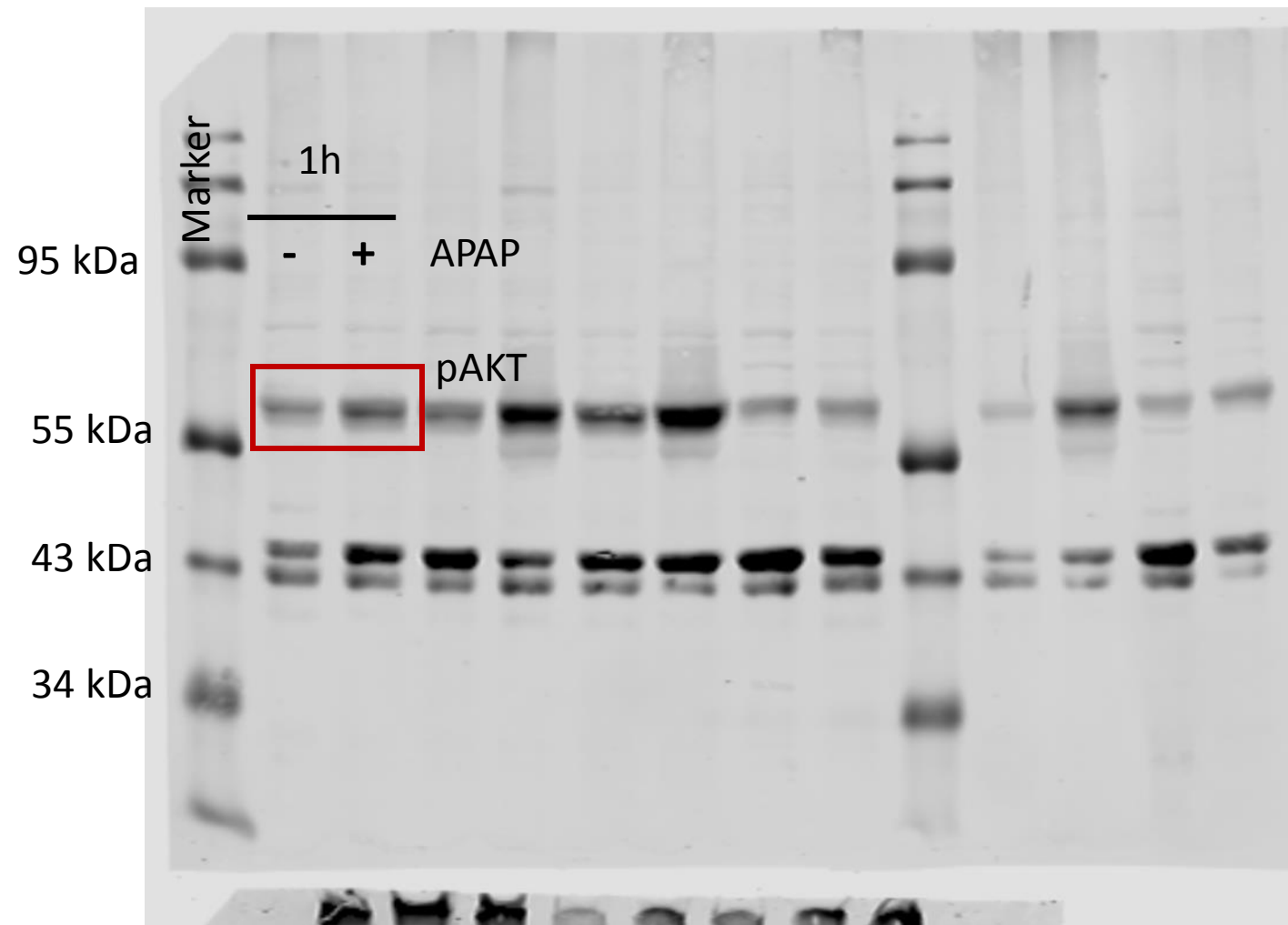

Marker

1h

- + APAP

AKT

Supplement: Figure 4—source data 1. [file elife-78540-fig4-data1.zip › Figure 4-source data/Figure 4A source data.pdf]

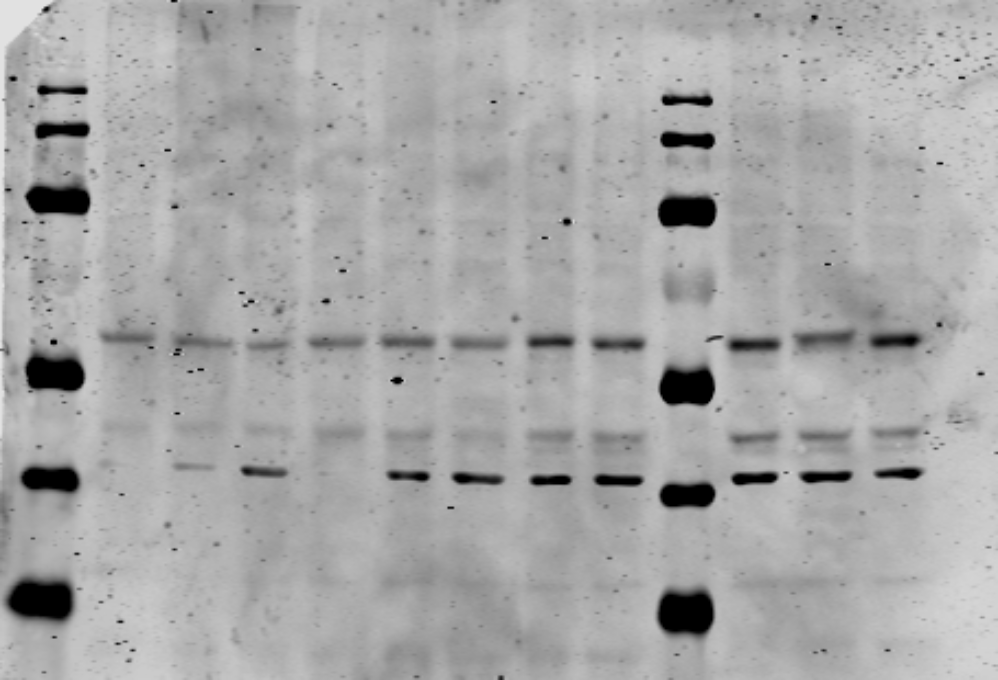

Supplement: Figure 4—source data 1. [file elife-78540-fig4-data1.zip › Figure 4-source data/Figure 4A/AKT reprobe.png]

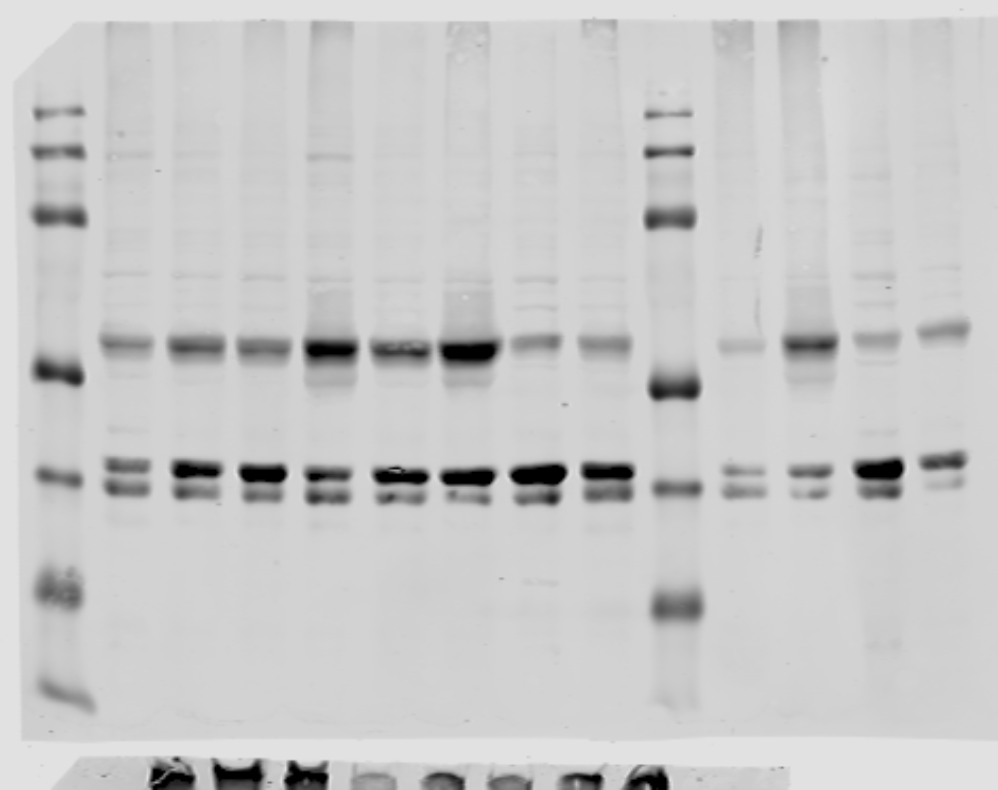

Supplement: Figure 4—source data 1. [file elife-78540-fig4-data1.zip › Figure 4-source data/Figure 4A/pAKT 3.png]

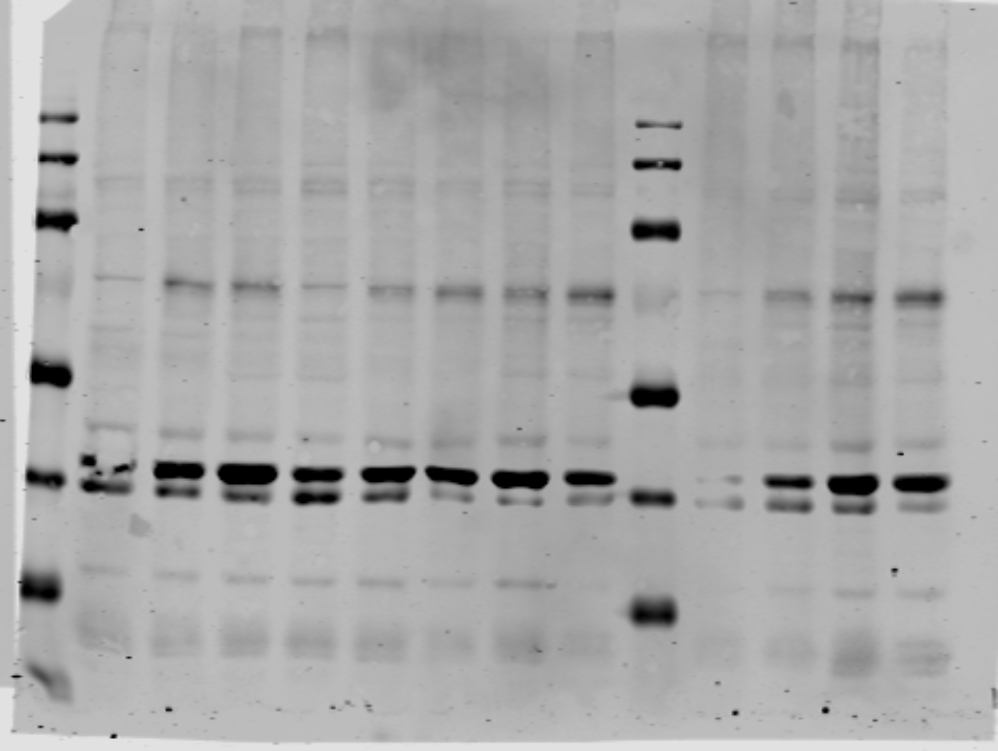

Supplement: Figure 4—source data 1. [file elife-78540-fig4-data1.zip › Figure 4-source data/Figure 4A/pYAP 3.png]

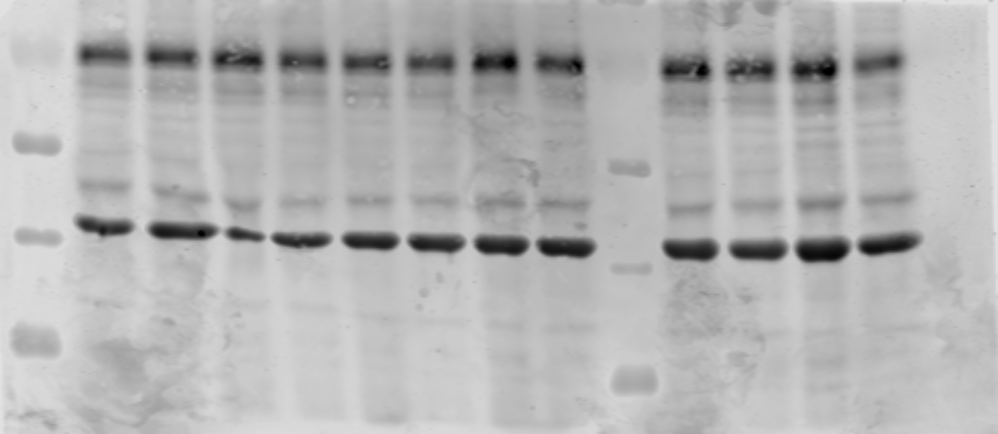

Supplement: Figure 4—source data 1. [file elife-78540-fig4-data1.zip › Figure 4-source data/Figure 4A/YAP actin.png]

Fig 4 C (pYAP + actin)

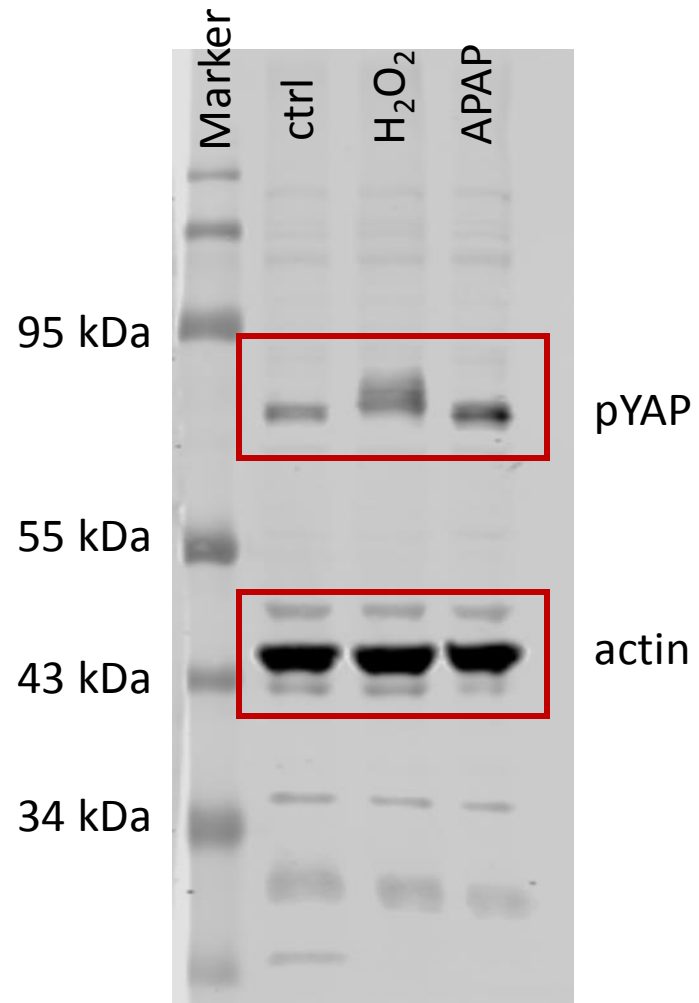

Fig 4 C (YAP)

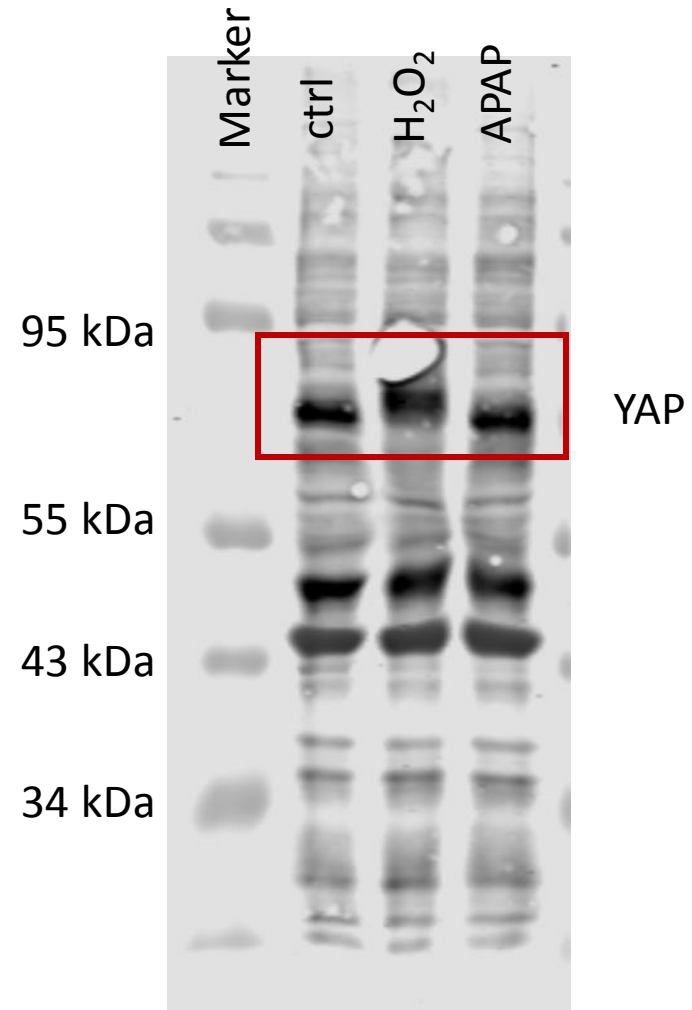

Fig 4 C (pAKT)

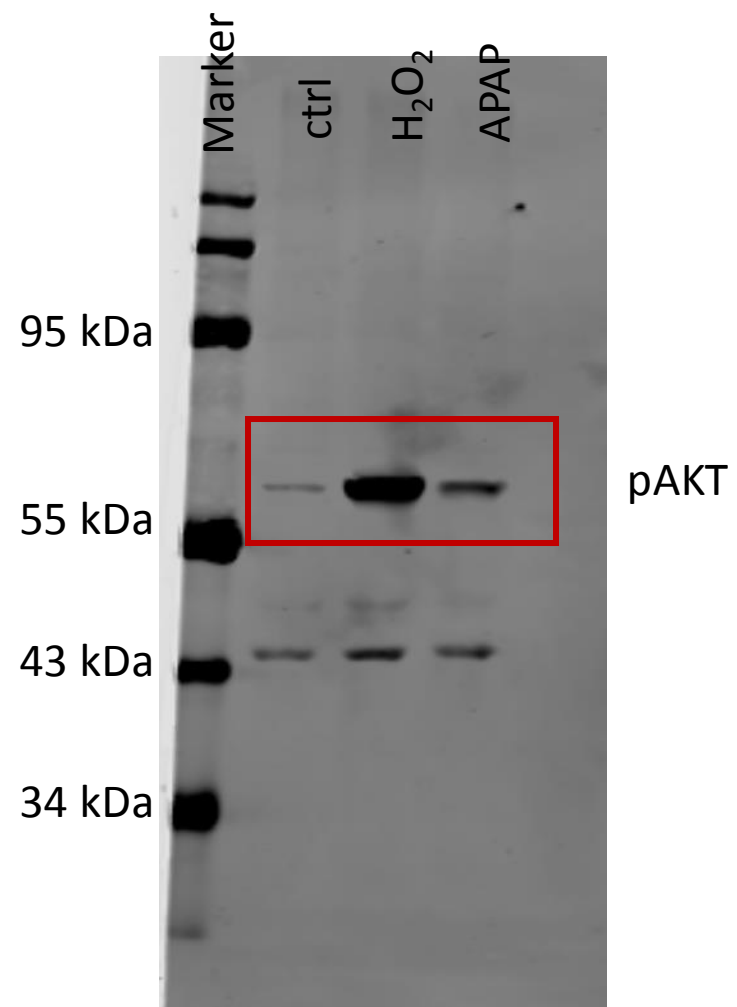

Fig 4 C (AKT)

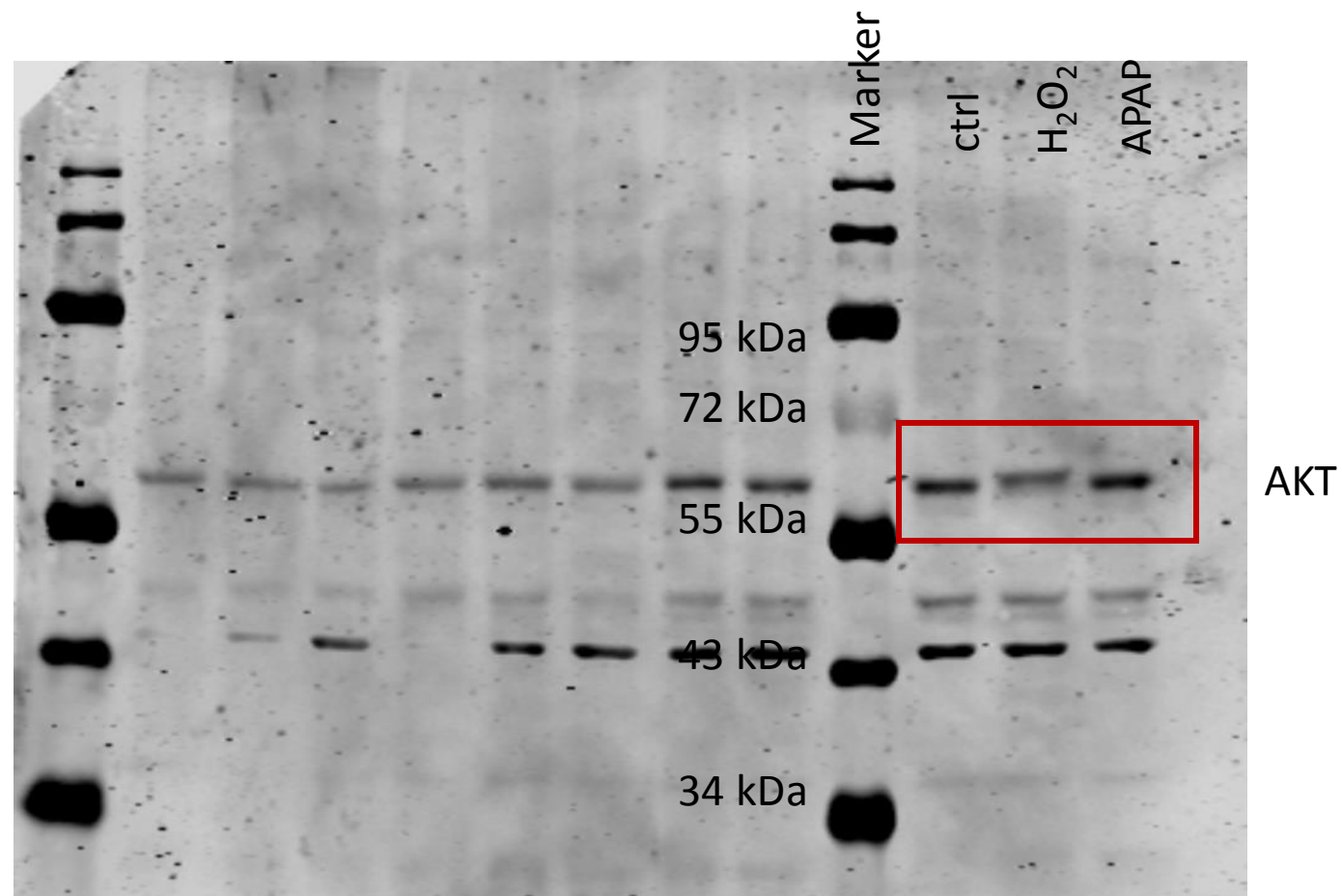

Supplement: Figure 4—source data 1. [file elife-78540-fig4-data1.zip › Figure 4-source data/Figure 4C source data.pdf]

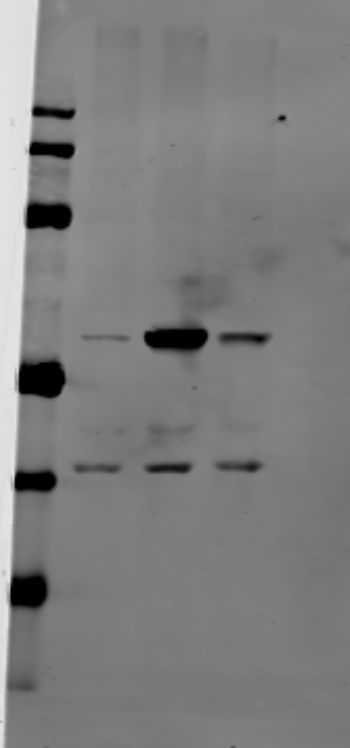

Supplement: Figure 4—source data 1. [file elife-78540-fig4-data1.zip › Figure 4-source data/Figure 4C/pAKT.png]

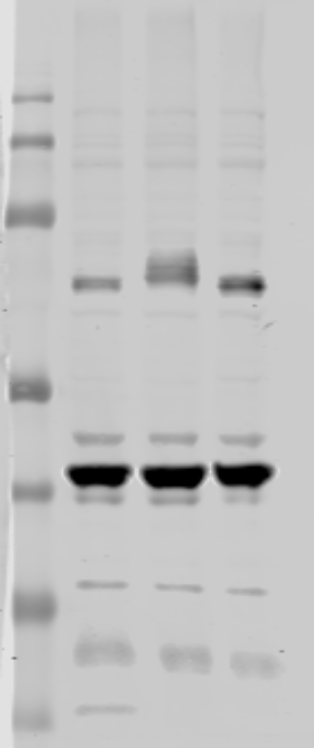

Supplement: Figure 4—source data 1. [file elife-78540-fig4-data1.zip › Figure 4-source data/Figure 4C/pYAP actin.png]

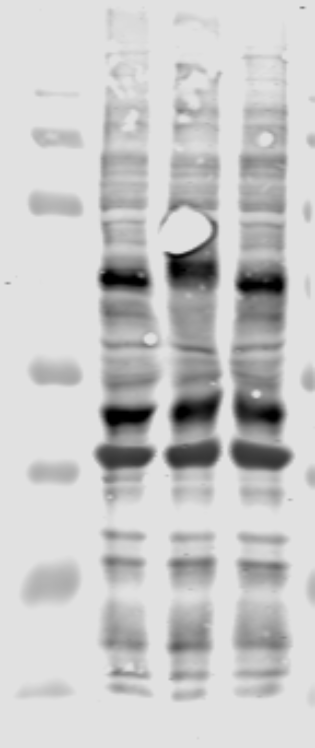

Supplement: Figure 4—source data 1. [file elife-78540-fig4-data1.zip › Figure 4-source data/Figure 4C/YAP.png]

Fig 4 D (pYAP)

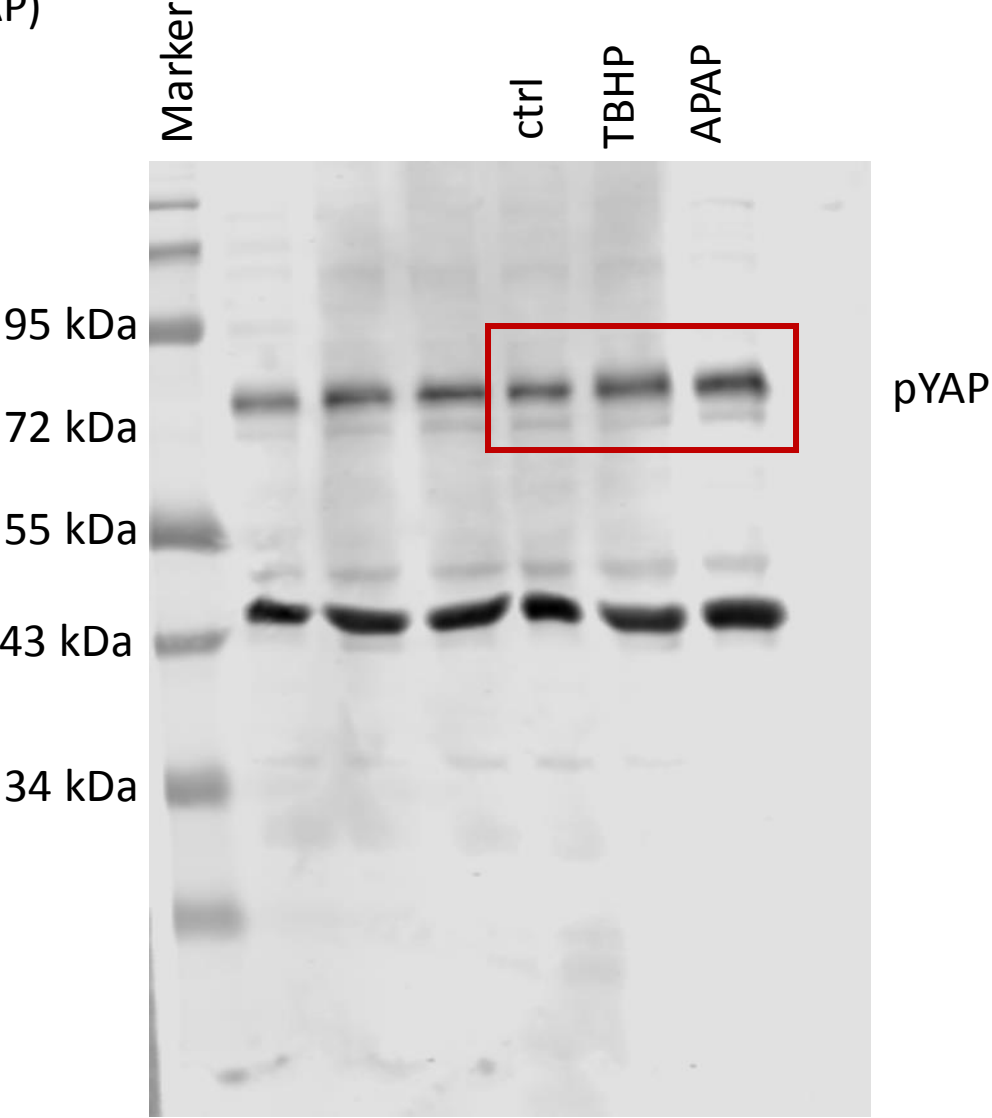

Fig 4 D (YAP)

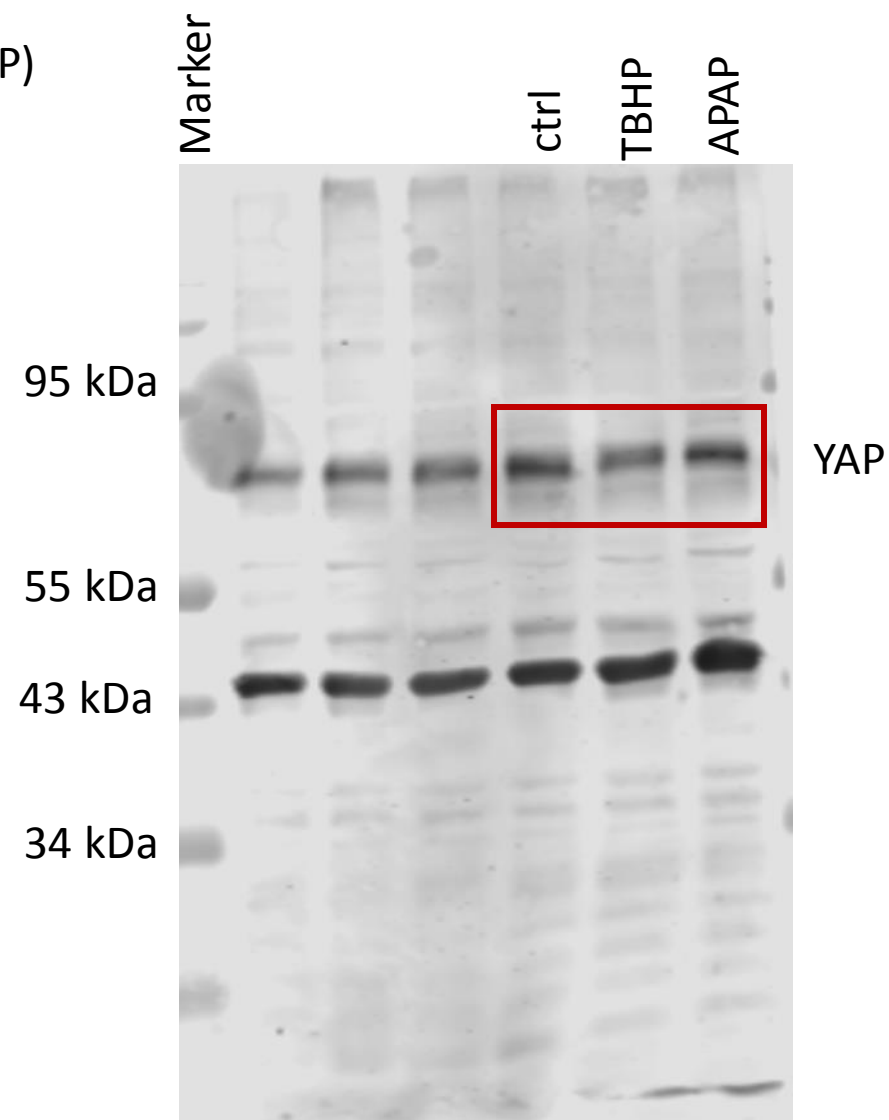

Fig 4 D (pAKT)

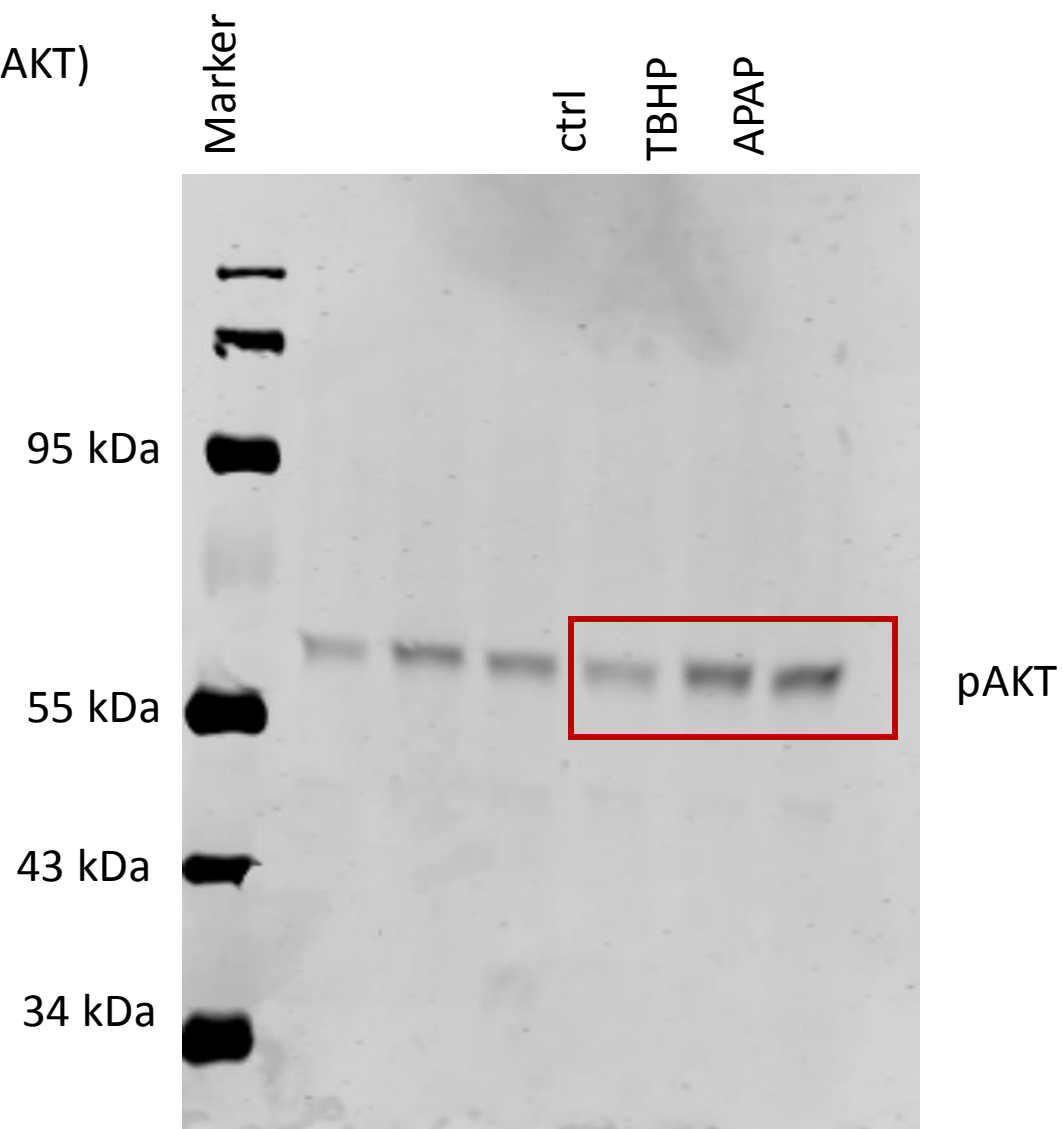

Fig 4 D (AKT + actin)

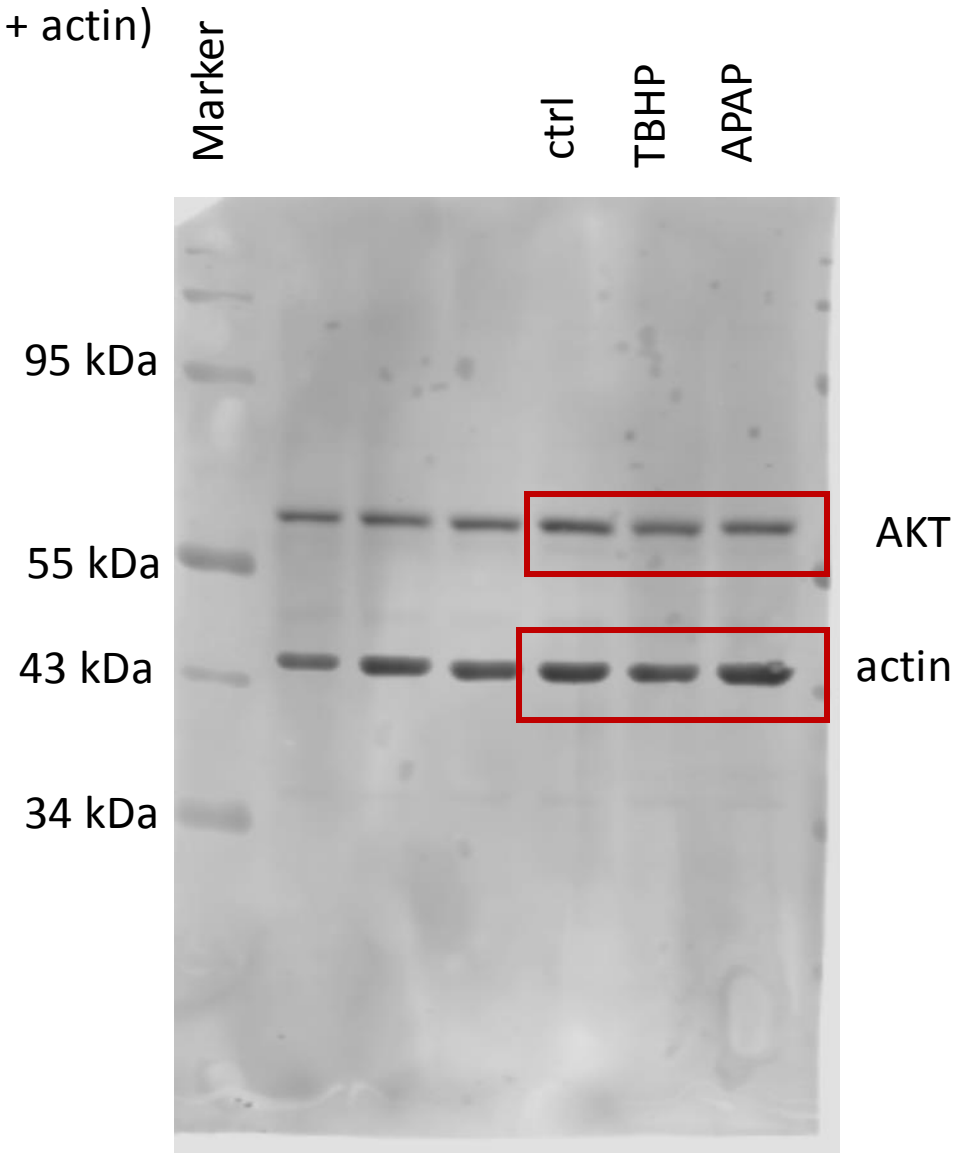

Supplement: Figure 4—source data 1. [file elife-78540-fig4-data1.zip › Figure 4-source data/Figure 4D source data.pdf]

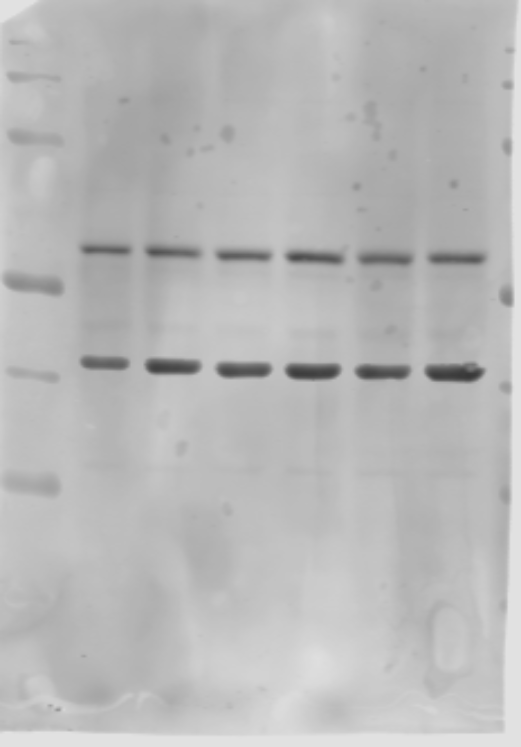

Supplement: Figure 4—source data 1. [file elife-78540-fig4-data1.zip › Figure 4-source data/Figure 4D/AKT actin.png]

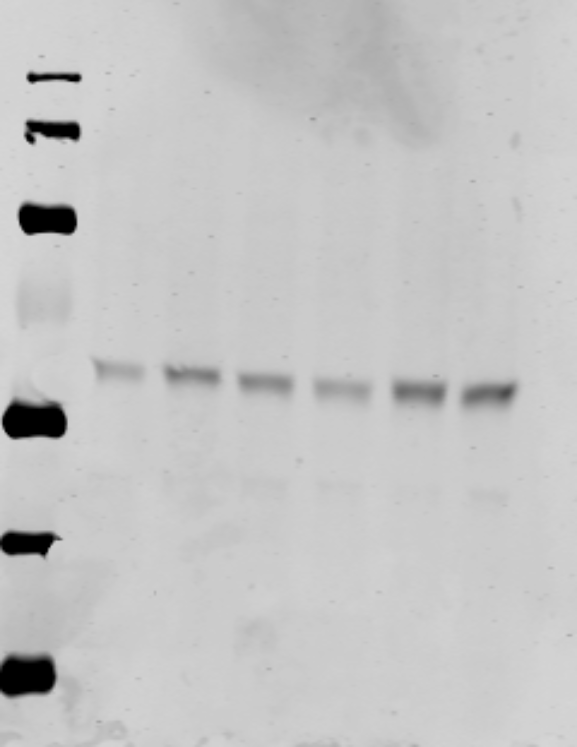

Supplement: Figure 4—source data 1. [file elife-78540-fig4-data1.zip › Figure 4-source data/Figure 4D/pAKT.png]

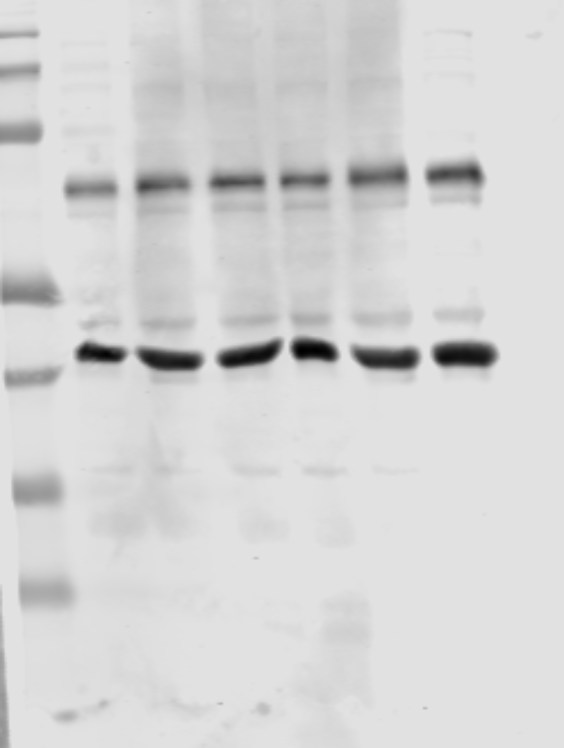

Supplement: Figure 4—source data 1. [file elife-78540-fig4-data1.zip › Figure 4-source data/Figure 4D/pYAP.png]

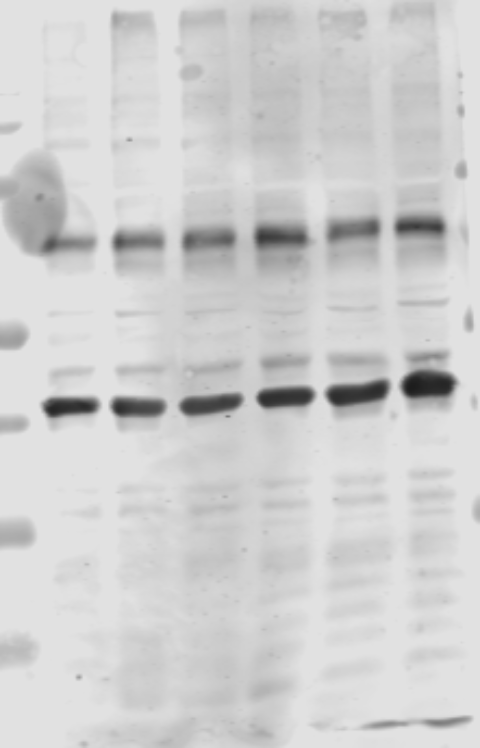

Supplement: Figure 4—source data 1. [file elife-78540-fig4-data1.zip › Figure 4-source data/Figure 4D/YAP.png]

Fig 4 E

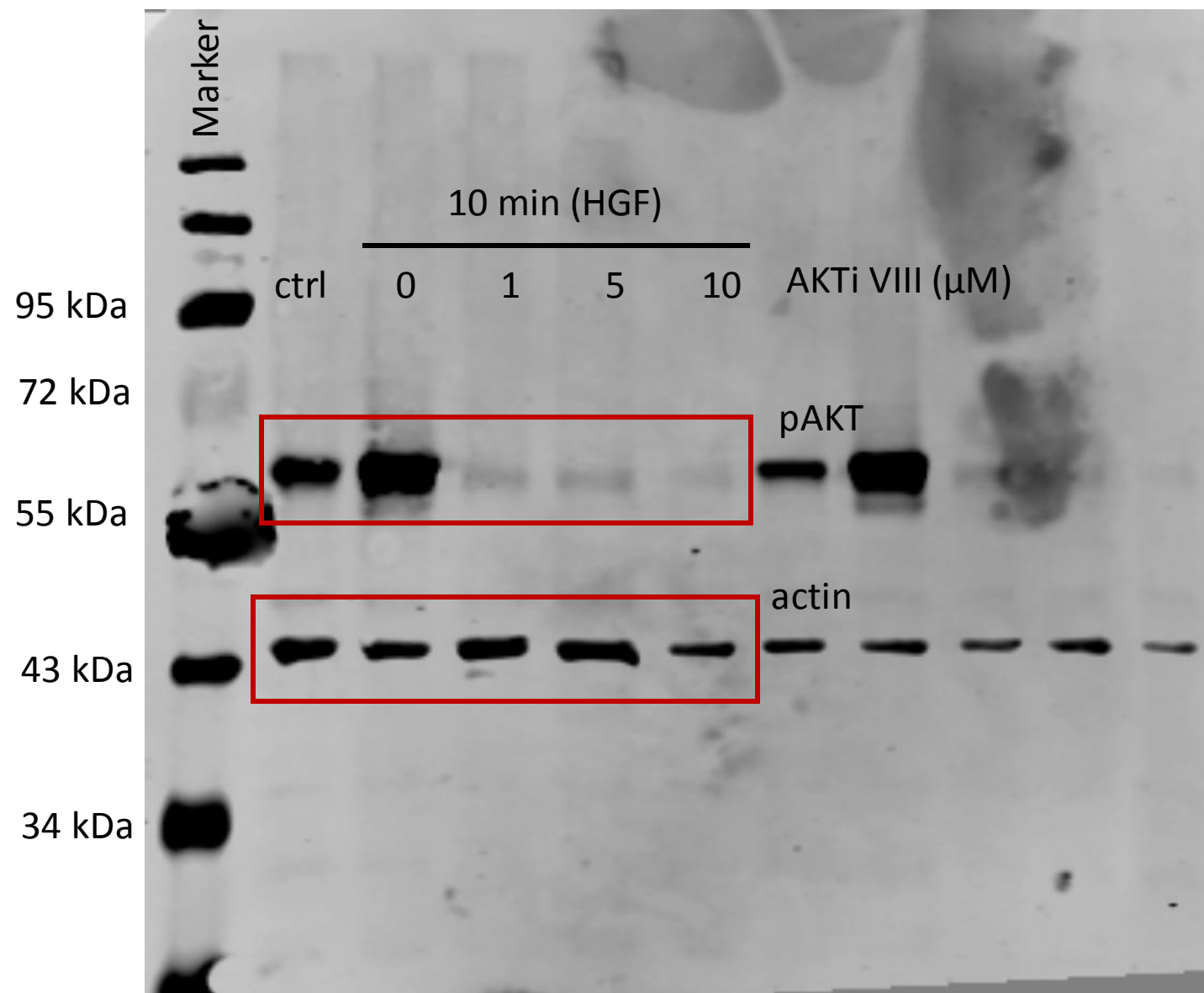

Fig 4 E

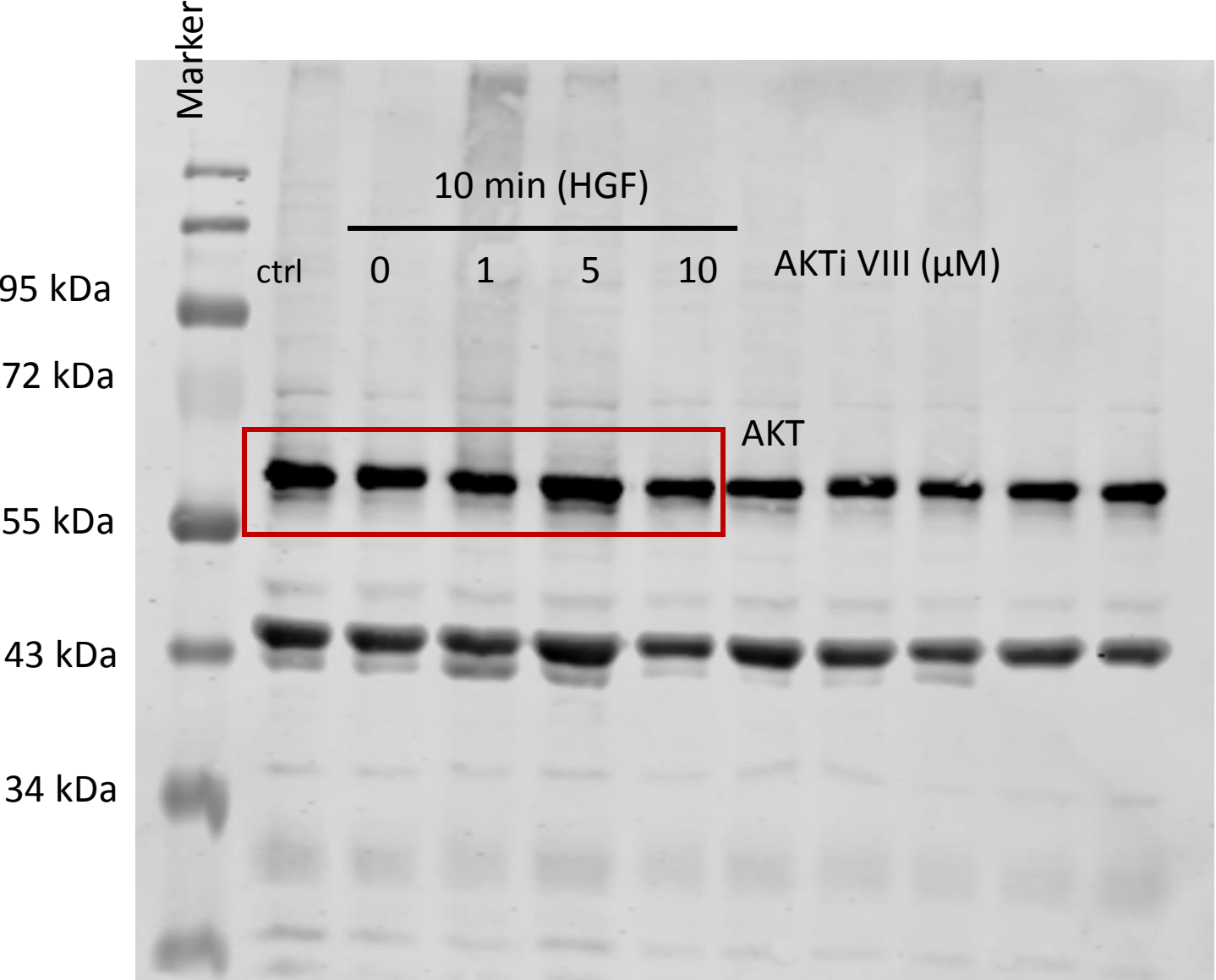

Fig 4 E

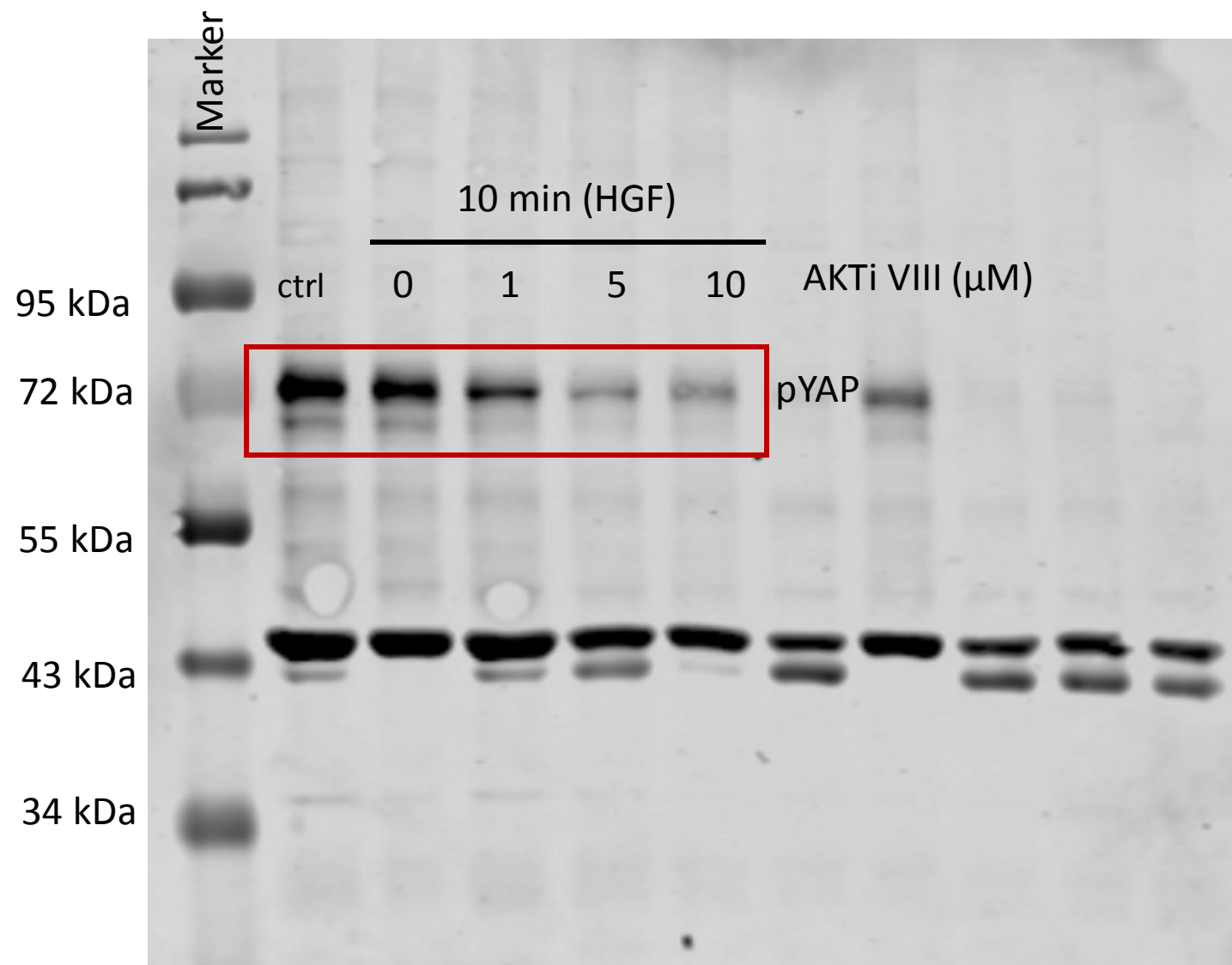

Fig 4 E

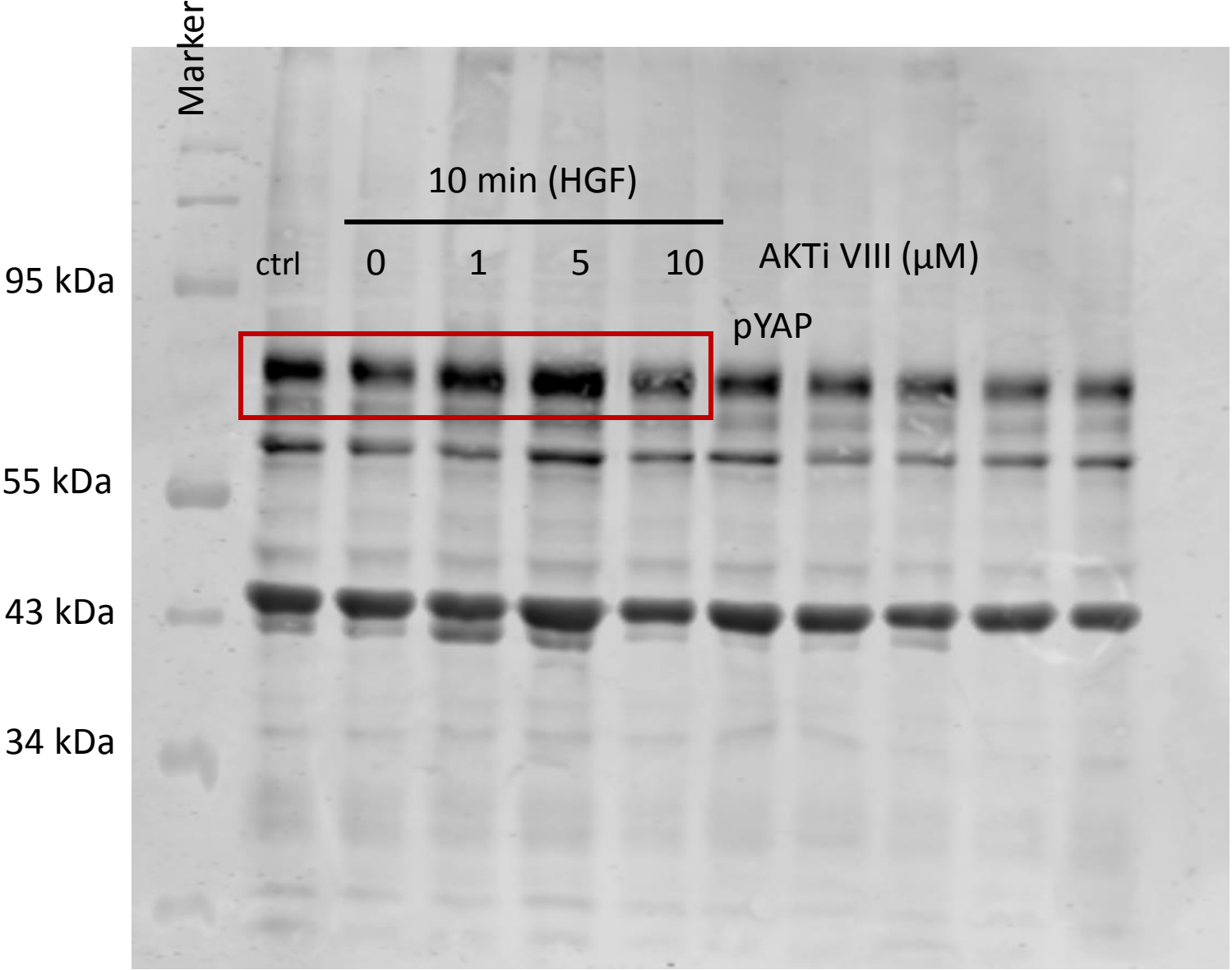

Supplement: Figure 4—source data 1. [file elife-78540-fig4-data1.zip › Figure 4-source data/Figure 4E source data.pdf]

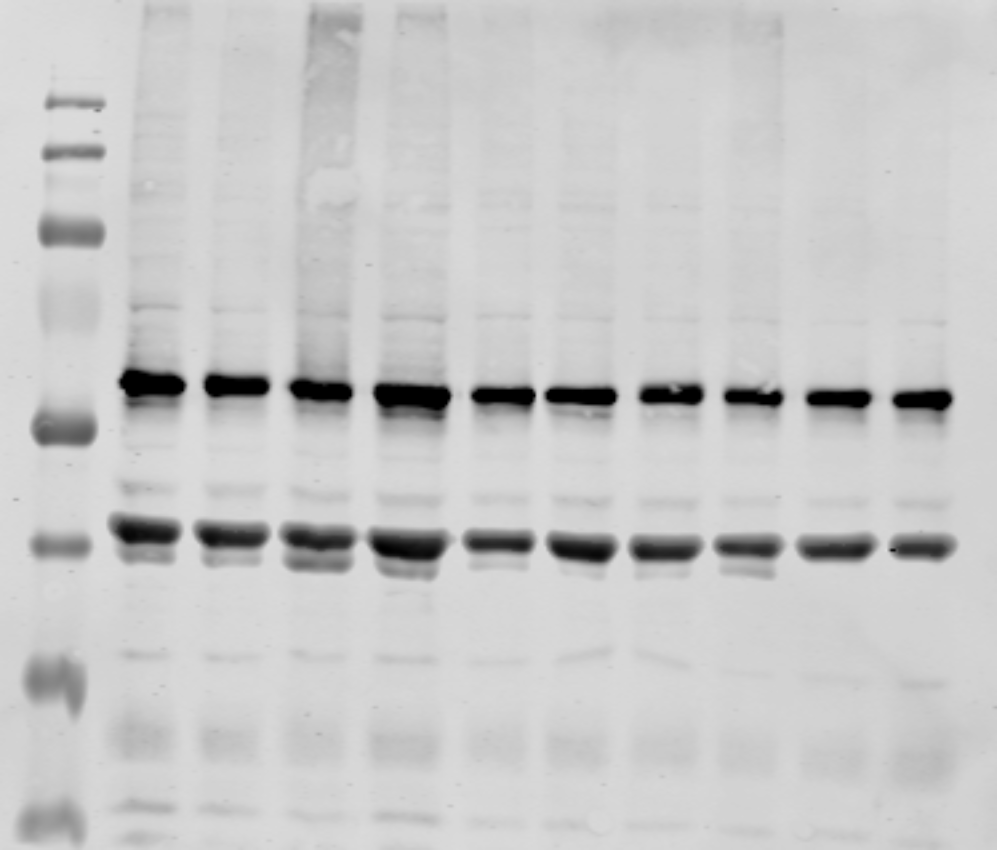

Supplement: Figure 4—source data 1. [file elife-78540-fig4-data1.zip › Figure 4-source data/Figure 4E/AKT.png]

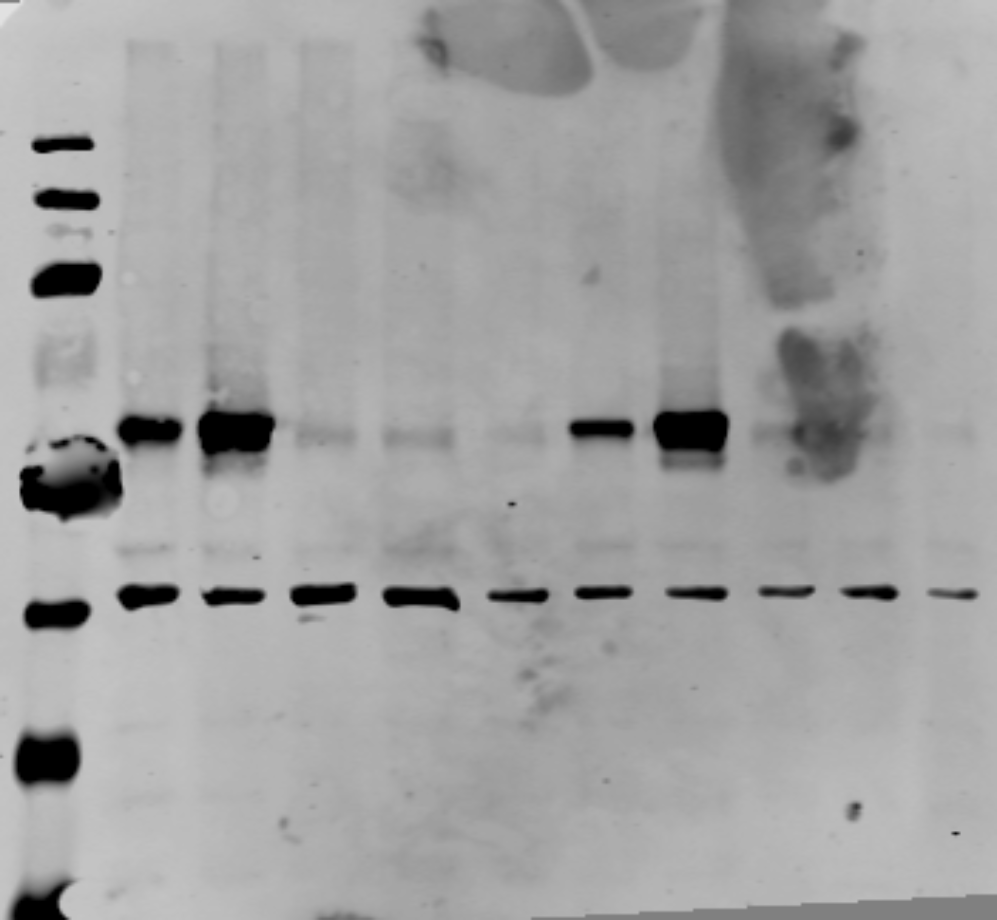

Supplement: Figure 4—source data 1. [file elife-78540-fig4-data1.zip › Figure 4-source data/Figure 4E/pAKT actin.png]

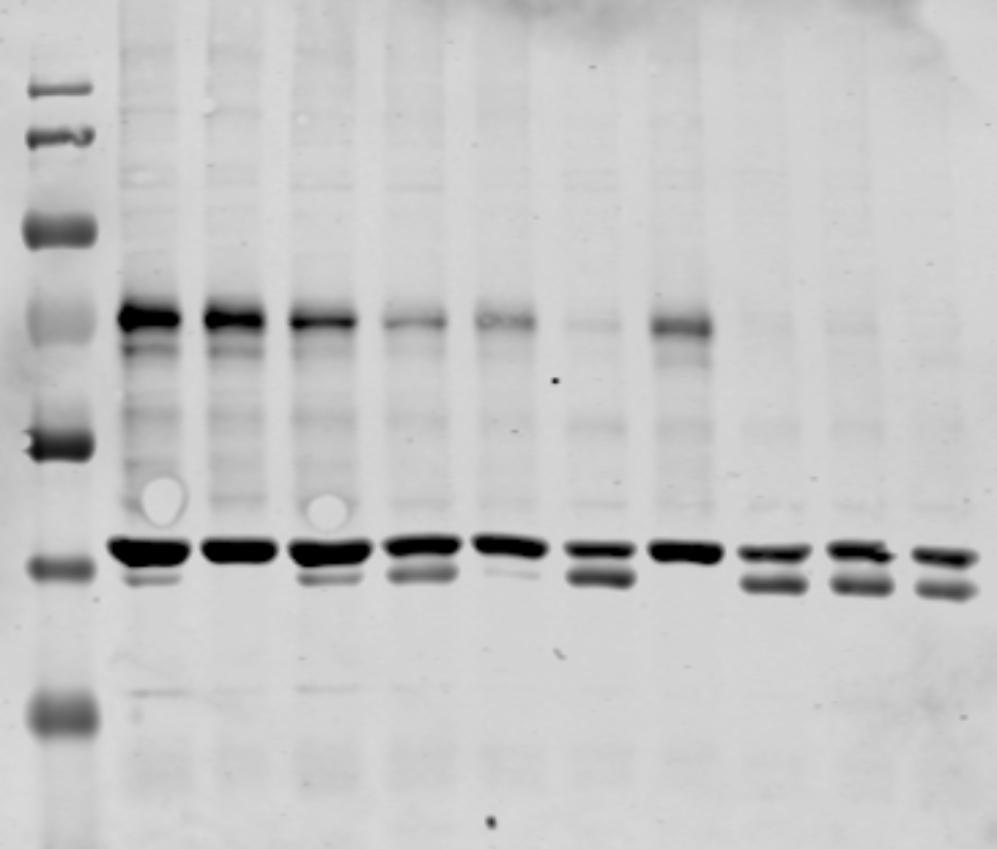

Supplement: Figure 4—source data 1. [file elife-78540-fig4-data1.zip › Figure 4-source data/Figure 4E/pYAP.png]

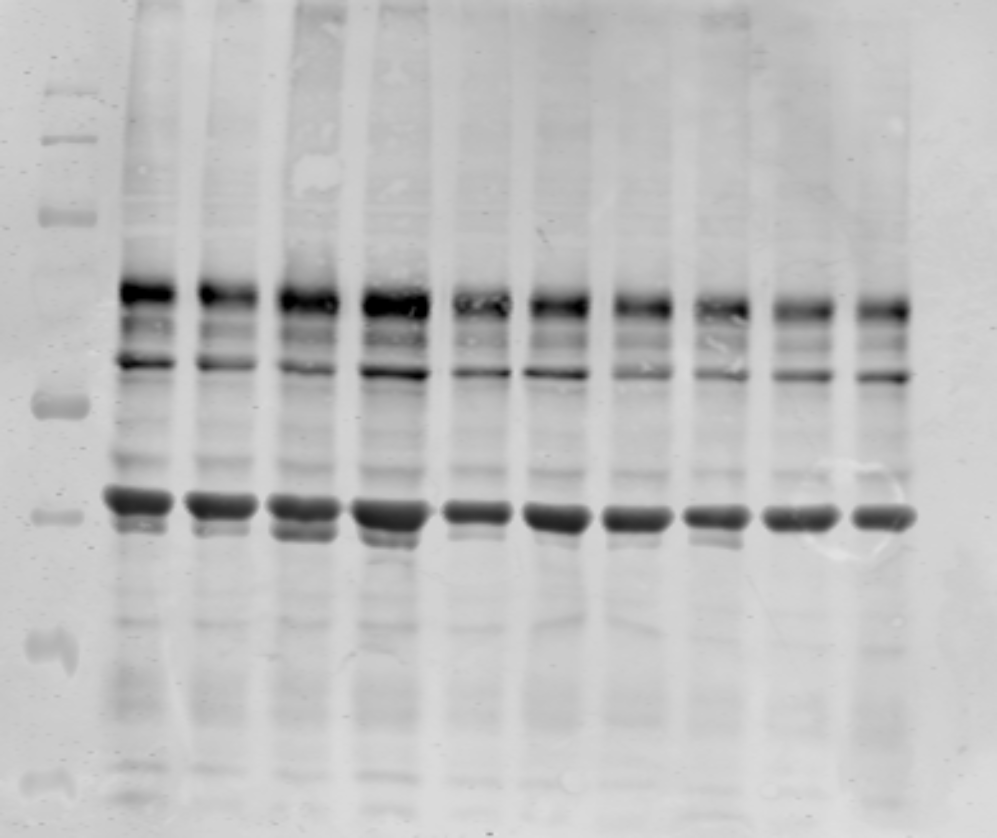

Supplement: Figure 4—source data 1. [file elife-78540-fig4-data1.zip › Figure 4-source data/Figure 4E/YAP (reprobed in AKT).png]

Fig 4F (pAKT)

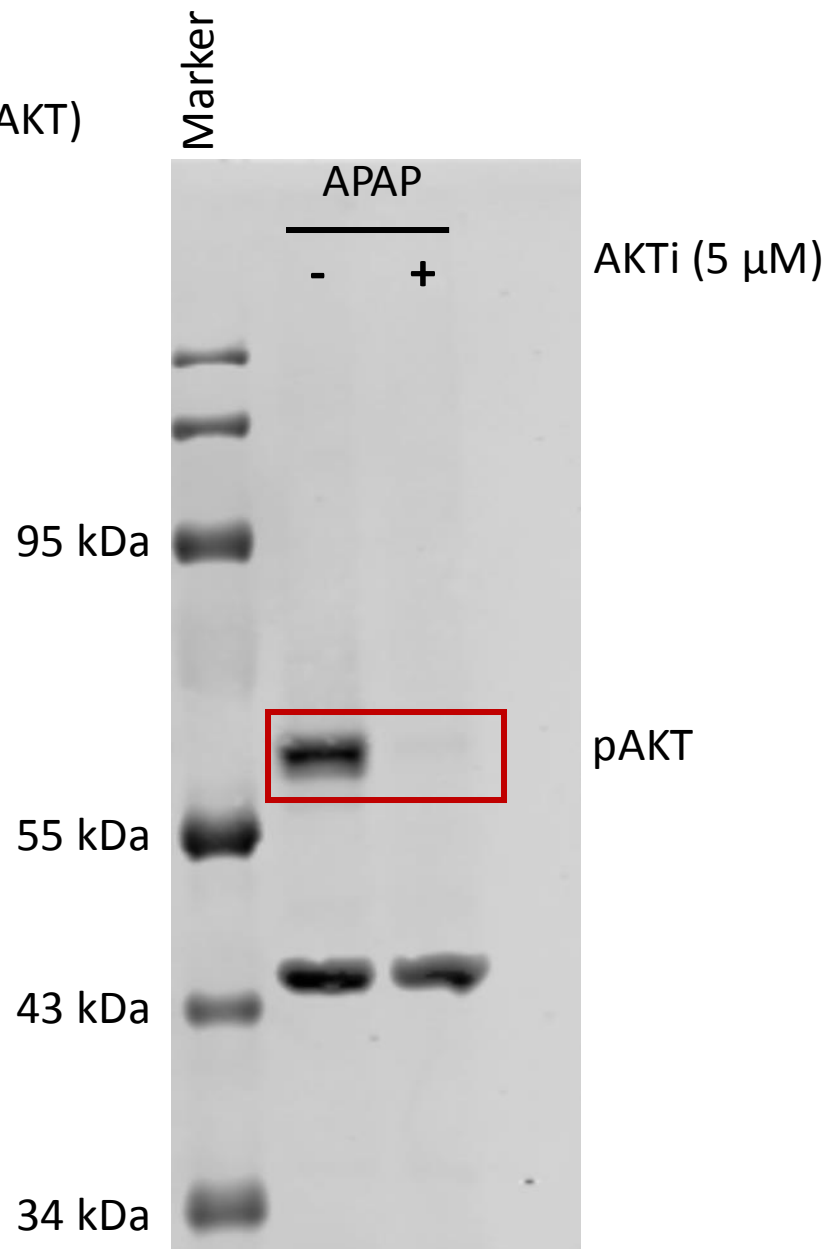

Fig 4F (AKT)

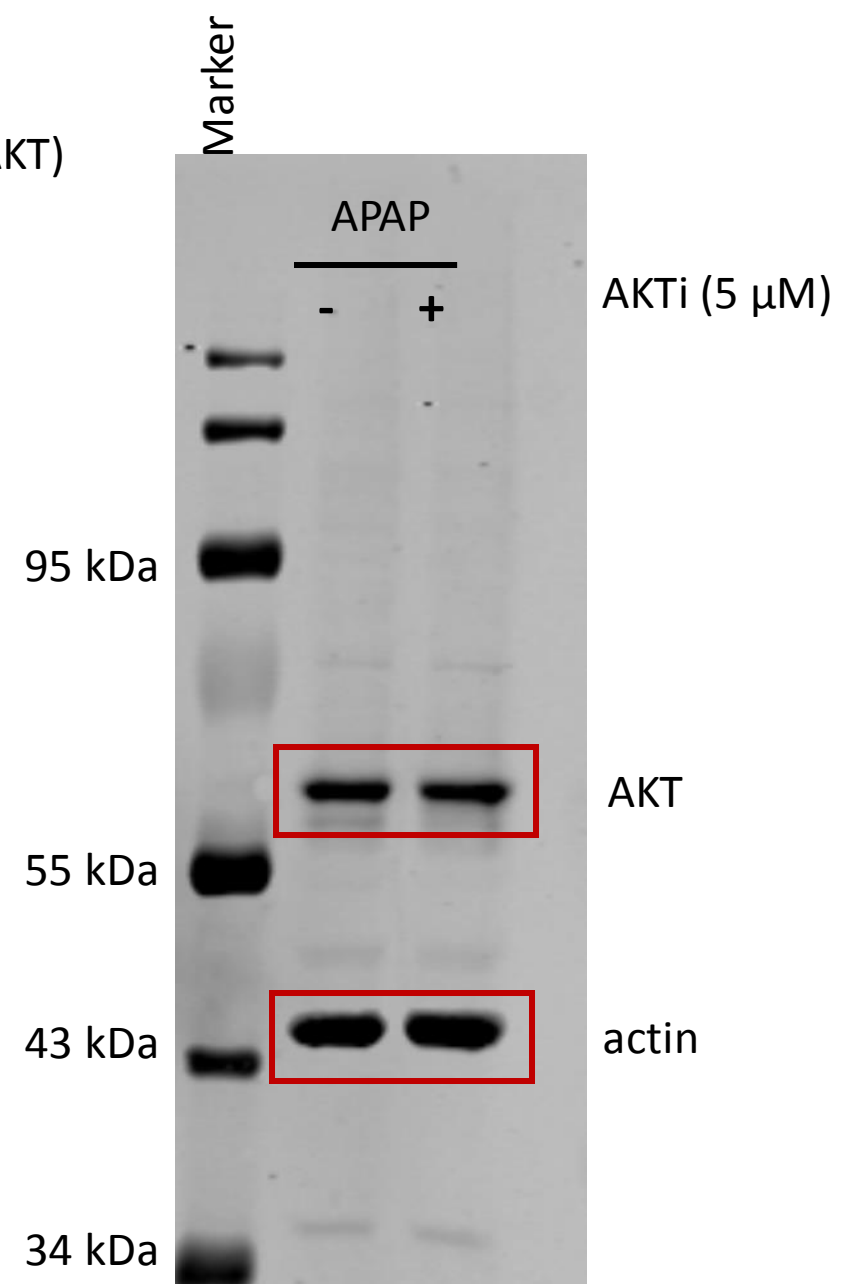

Fig 4F (pYAP)

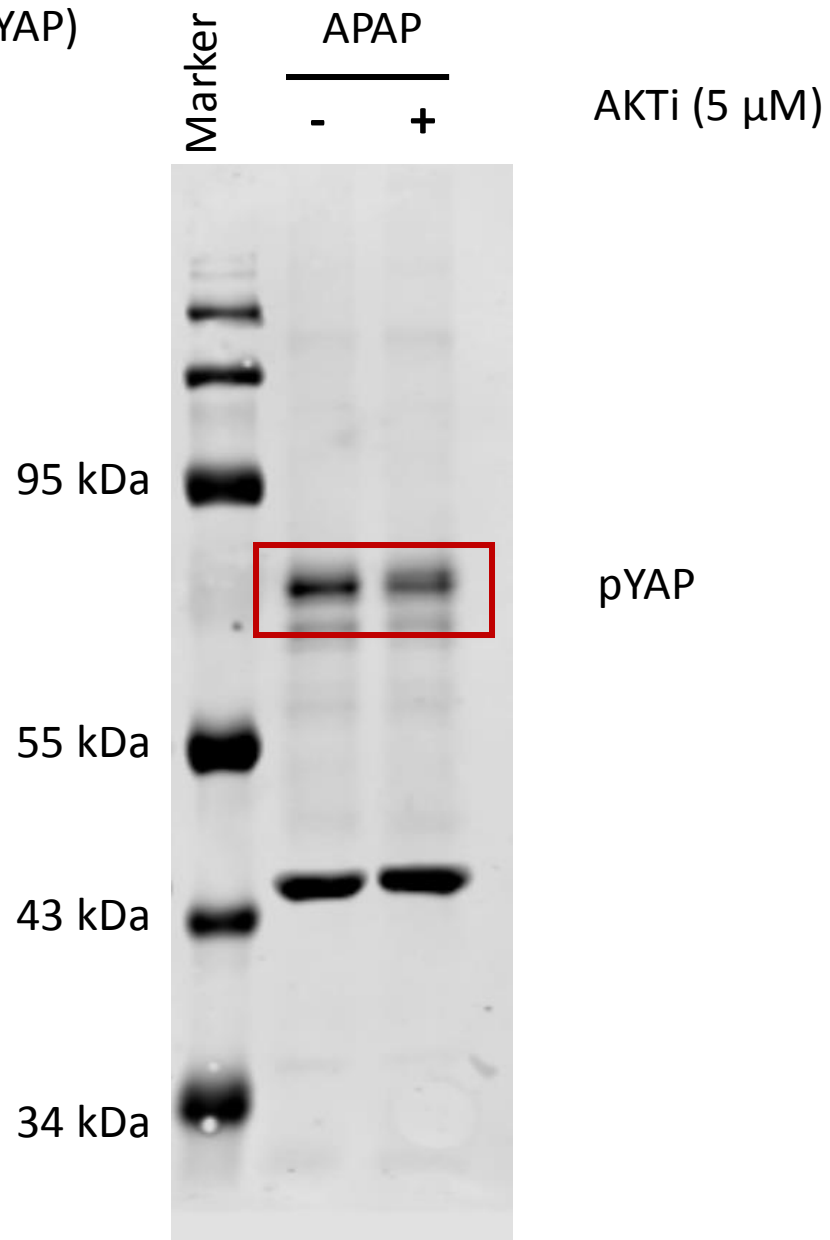

Fig 4F (YAP)

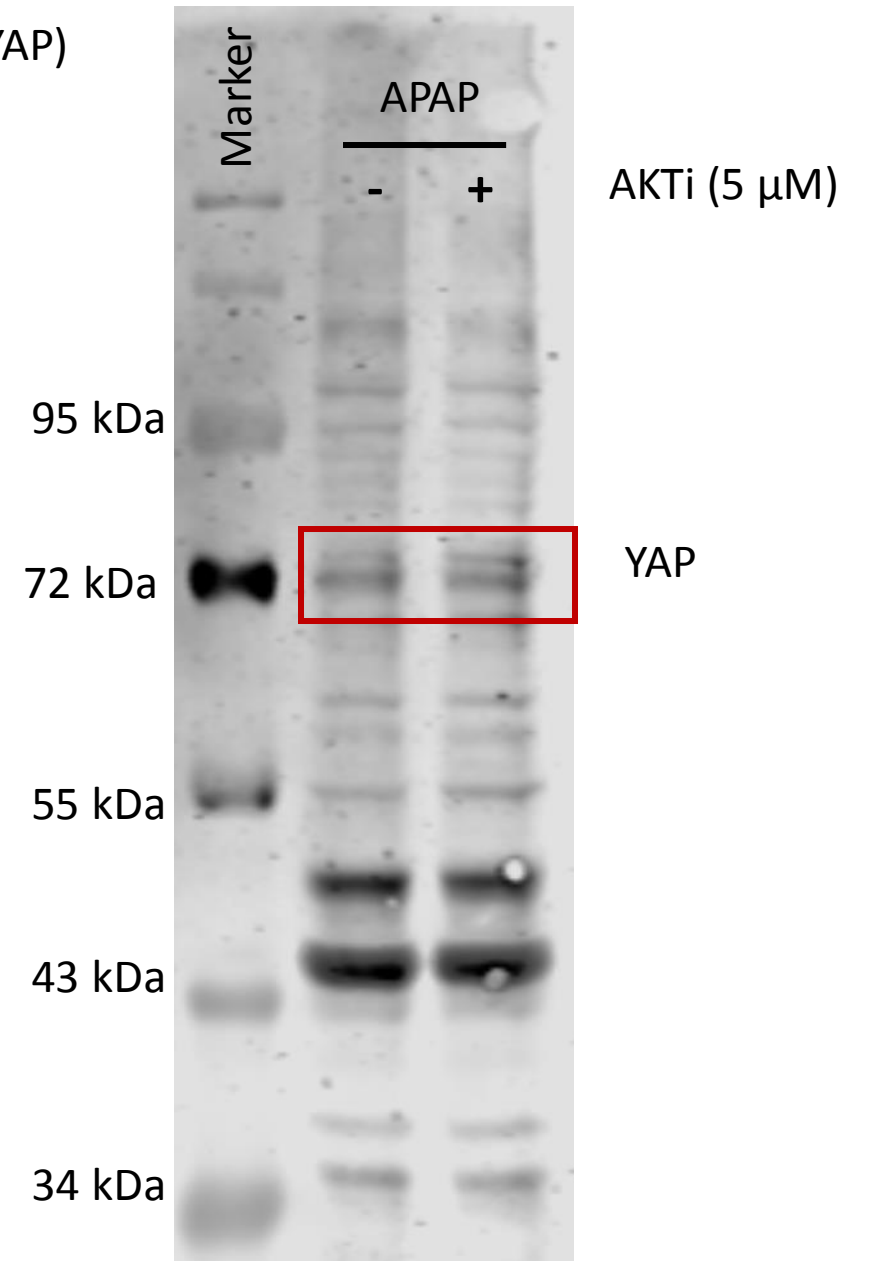

Supplement: Figure 4—source data 1. [file elife-78540-fig4-data1.zip › Figure 4-source data/Figure 4F source data.pdf]

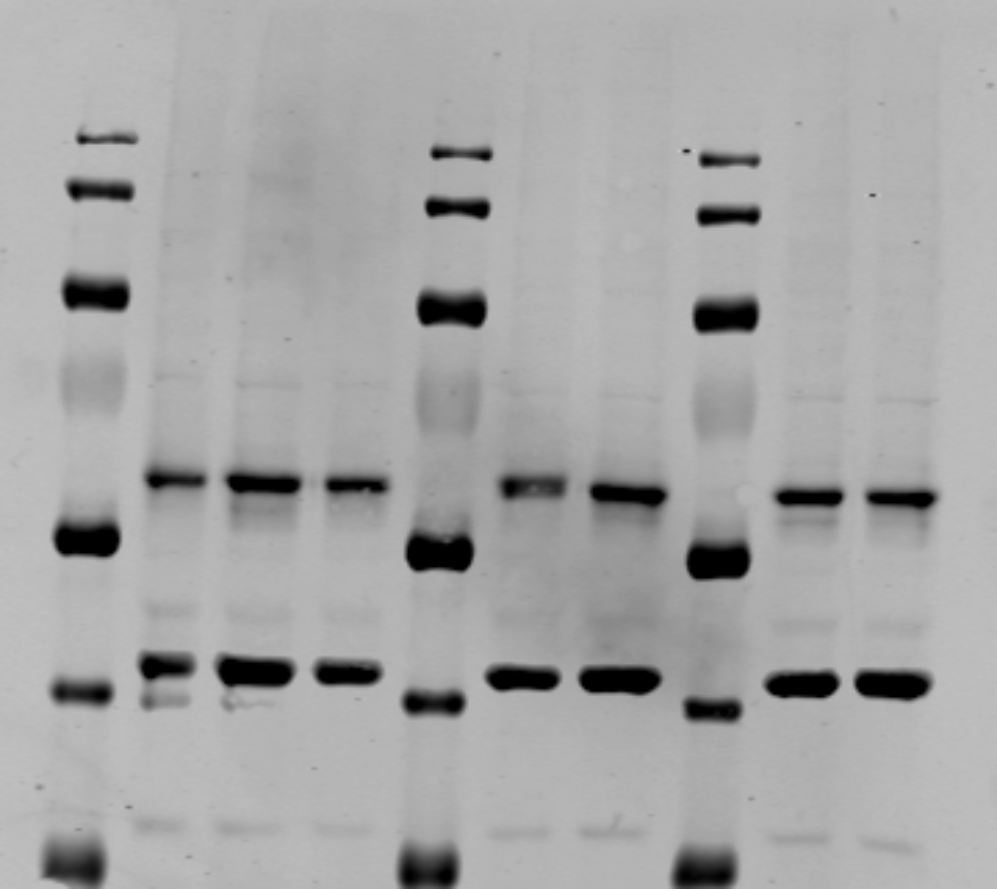

Supplement: Figure 4—source data 1. [file elife-78540-fig4-data1.zip › Figure 4-source data/Figure 4F/AKT actin.png]

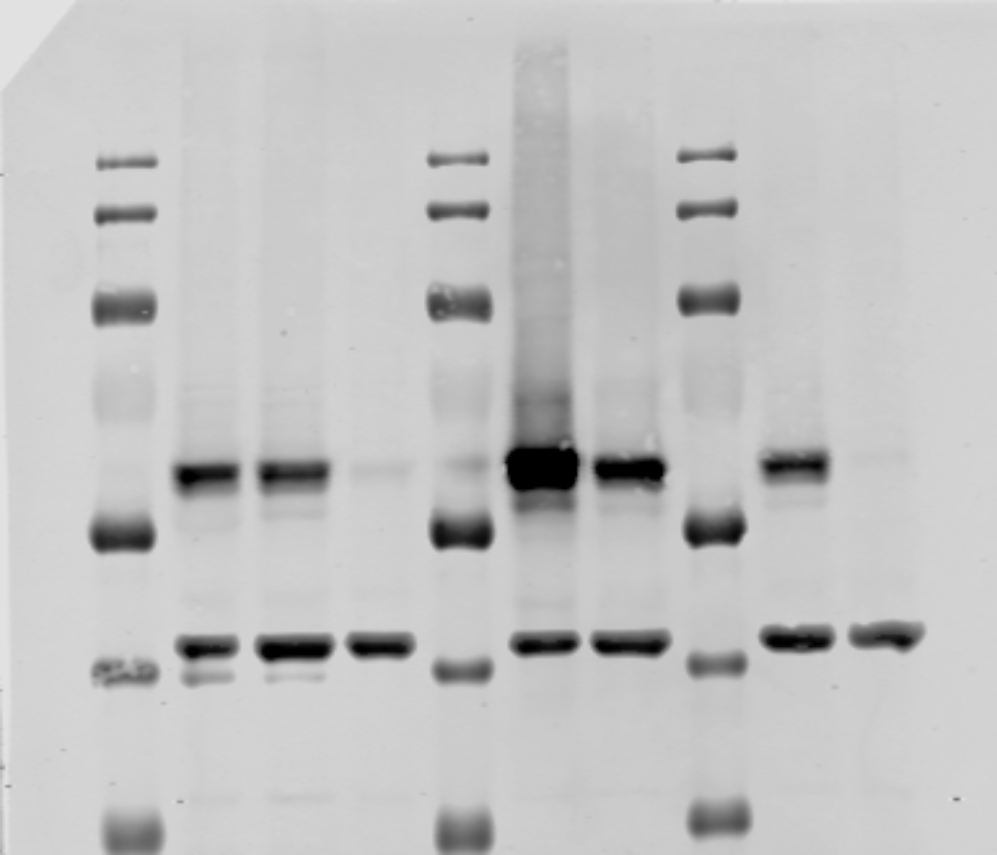

Supplement: Figure 4—source data 1. [file elife-78540-fig4-data1.zip › Figure 4-source data/Figure 4F/pAKT.png]

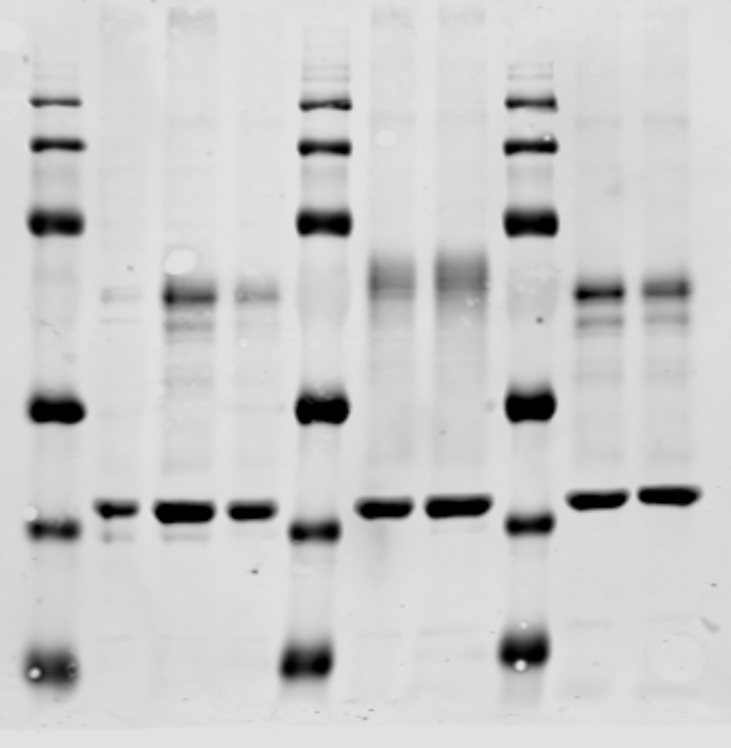

Supplement: Figure 4—source data 1. [file elife-78540-fig4-data1.zip › Figure 4-source data/Figure 4F/pYAP.png]

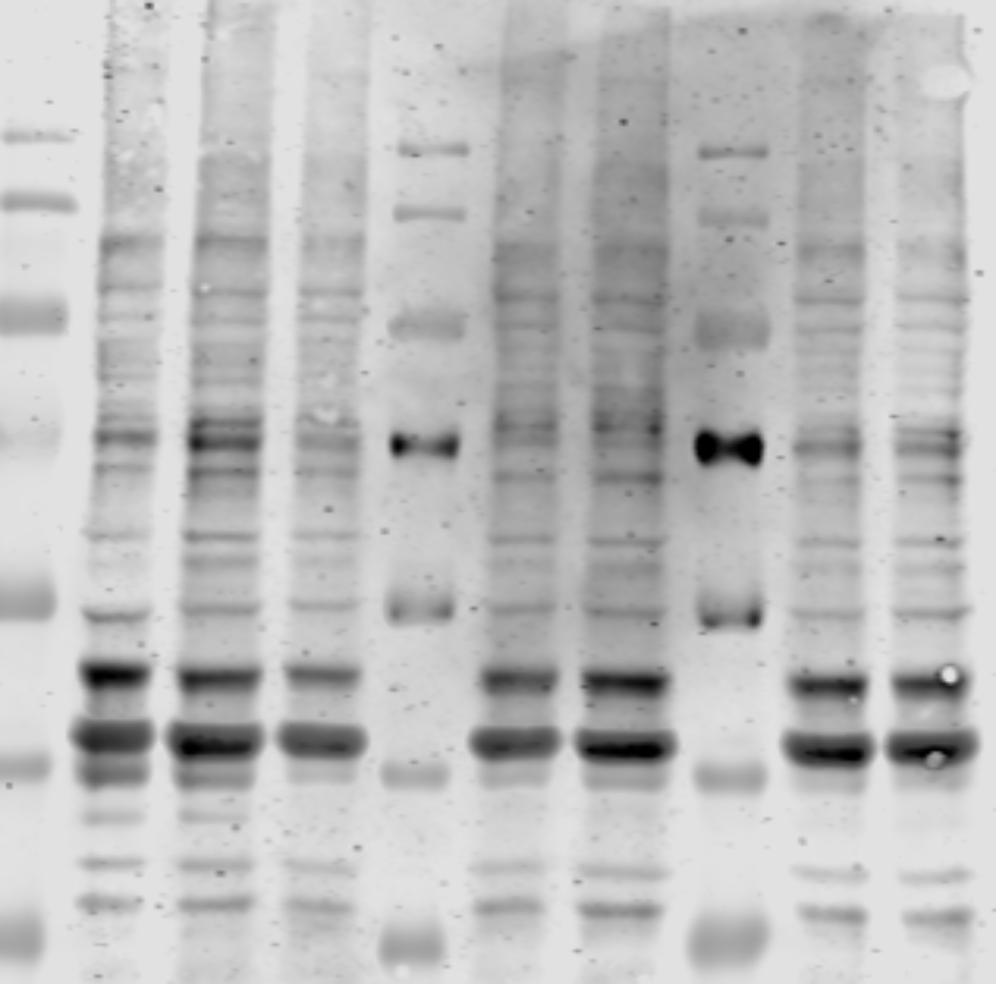

Supplement: Figure 4—source data 1. [file elife-78540-fig4-data1.zip › Figure 4-source data/Figure 4F/YAP.png]
